# Rhythmic cueing, dance, resistance training, and Parkinson's disease: A systematic review and meta-analysis

**DOI:** 10.3389/fneur.2022.875178

**Published:** 2022-08-09

**Authors:** Claire Chrysanthi Karpodini, Petros C. Dinas, Efthalia Angelopoulou, Matthew A. Wyon, Aline Nogueira Haas, Maria Bougiesi, Sokratis G. Papageorgiou, Yiannis Koutedakis

**Affiliations:** ^1^Sport and Physical Activity Research Centre, Faculty of Education, Health and Wellbeing, University of Wolverhampton, Wolverhampton, United Kingdom; ^2^Functional Architecture of Mammals in their Environment Laboratory, Department of Physical Education and Sport Science, University of Thessaly, Volos, Greece; ^3^First Department of Neurology, Medical School, National and Kapodistrian University of Athens, Eginition University Hospital, Athens, Greece; ^4^School of Physical Education Physiotherapy and Dance, Federal University of Rio Grande do Sul (UFRGS), Porto Alegre, Brazil

**Keywords:** Parkinson's disease, rhythm, dance, strength, systematic review, meta-analysis

## Abstract

**Objectives:**

The aim of the present systematic review and meta-analysis was to synthesize evidence associated with the functional and clinical effectiveness of rhythmic cueing, dance, or resistance training (RT) on motor and non-motor parameters in Parkinson's Disease patients, and to provide a comparative perspective not offered by existing systematic reviews.

**Methodology:**

Eligibility criteria for selecting studies retained no restrictions in methodological design and included interventions of rhythmic cueing, dance, RT, and measurements of motor and non-motor parameters. Animal studies, reviews, editorials, conferences, magazines, and gray literature articles were excluded. Two independent investigators searched Cochrane Library, Medline, PubMed, and SPORTDiscus from the date of their inception until 1 June 2021. The ROBINS-I tool was employed for the non-randomized controlled trials, and the updated for Risk of Bias 2 tool of Cochrane Library used for randomized controlled trials. For meta-analyses, the RevMan 5.4.13 software was used. For incompatible meta-analysis studies, a narrative data synthesis was conducted.

**Results:**

A total of 49 studies included in the systematic review involving 3767 PD participants. Meta-analyses revealed that rhythmic cueing training assists gait velocity (*p* = 0.01), stride length (*p* = 0.01), and motor symptoms (*p* = 0.03). Similarly, dance training benefits stride length (*p* = 0.05), lower extremity function-TUG (*p* = 0.01), and motor symptoms (*p* = 0.01), whilst RT improves lower extremity function-TUG (*p* = 0.01), quality of life (*p* = 0.01), knee flexion (*p* = 0.02), and leg press (*p* = 0.01). Subgroup analyses have shown non-significant differences in gait velocity (*p* = 0.26), stride length (*p* = 0.80), functional mobility-TUG (*p* = 0.74), motor symptoms-UPDRS-III (*p* = 0.46), and quality of life-PDQ39 (*p* = 0.44).

**Conclusion:**

Rhythmic cueing, dance, or RT positively affect the examined outcomes, with rhythmic cueing to be associated with three outcomes (Gait, Stride, and UPDRS-III), dance with three outcomes (TUG, Stride, and UPDRS-III), and RT with two outcomes (TUG and PDQ-39). Subgroup analyses confirmed the beneficial effects of these forms of exercise. Clinicians should entertain the idea of more holistic exercise protocols aiming at improving PD manifestations.

International Prospective Register of systematic reviews (PROSPERO) (registration number: CRD42020212380).

## Introduction

Parkinson's disease (PD) is a progressive neurodegenerative disorder, which is mainly characterized by the loss of dopaminergic neurons in the substantia Nigra pars compacta (SNpc) of the midbrain and the accumulation of Lewy bodies and Lewy neuritis (1). Being the second most common neurodegenerative disorder (2), PD affects approximately 10 million people worldwide (3). It is estimated that by 2040 this number will increase over 12 million (4), with aging, as well as genetic and environmental factors contributing to its development (5). Physical exercise accompanied by healthy lifestyle has been shown to exert beneficial effects on the progression of the disease [(6–8)].

Some of the most common non-motor manifestations of PD include sleeping disorders, cognitive impairment (e.g., difficulties in concentrating, learning, remembering, and thinking), anxiety, depression, and lack of motivation (9). Motor manifestations include resting tremor, bradykinesia, freezing of gait, rigidity, and postural impairment. In PD, nigrostriatal degeneration resulting in basal ganglia dysfunction is critically associated with impaired synchronization of regular and periodical movement patterns (10, 11).

Auditory cues are beats that indicate a rhythmic schema, which usually consists of a monotonous tapping. Auditory cues can be any kind of rhythmic stimulation (12), while all beats are by default strong (13). For instance, the use of voice for counting, or syllabi (ya, ta, ta), or use of a tambourine or a metronome, or to move according to the meter of a music piece i.e., 2/4 or 4/4 time. When rhythmic schema is established, it can continue to exist in the listener's mind even when the source of rhythm is paused (13, 14). People usually synchronize their actions through an innate rhythmic entrainment (13), and in a healthy brain, this procedure is related to subcortico-thalamo-cortical network including the pre-supplementary and supplementary motor areas, basal ganglia, and cerebellum (12). Basal ganglia, and especially the putamen, is critically implicated in the sequencing of rhythmic stimuli, and potentially the ‘feeling of the beat’ (13). Acoustic cues may enhance the connectivity between auditory perception and movement, since rhythm enables the activation of neural circuits associated with motor processing (13). Given that PD patients display difficulties in performing automatized movements, the use of external cues appears to be beneficial (15). Rhythm, as a form of external cue, therefore, seems to reduce the dependence on deficient automatized processes (16) that characterize PD pathophysiology, since movement could be synchronized to the regular expectation of a beat (13).

Indeed, a systematic review, containing 50 studies with 1,892 PD participants, revealed the beneficial effects of external rhythmical cues on gait (17). However, another systematic review underlined the lack of consistency in studies with rhythmic auditory stimulation in most components such as participants, exercise intervention, duration, or design (12).

According to Malloch and Trevarthen (18), rhythm usually stands between music and dance, interacts between music and movement/dance, and forms the first step toward musicality. Dance itself is an activity as old as human civilization (19, 20), and in ancient Greece, it was used to improve or maintain health, especially in older people (21). Studies in dance displayed different methodological characteristics, such as type of dance, duration of intervention, and group comparisons (22). However, recent literature indicates that dance can improve selected motor and non-motor elements, such as gait, cognition, quality of life (QoL), and mood (22, 23), as it increases - brain-derived neurotrophic factor (BDNF) levels that, *inter alia*, trigger dopamine's production, an important aspect of PD pathophysiology (22, 24, 25). In addition, neurophysiological evidence via functional magnetic resonance imaging (fMRI) has shown that dance is associated with enhanced functional connectivity between premotor cortex and basal ganglia, while electroencephalogram (EEG) studies have demonstrated that Tango might alter muscle synergy during balance and walking testing (26). It has been found that dance provides environmental enrichment that positively affects social and emotional states by stimulating diverse sensory functions during dancing, such as audition, vision, proprioception and tactile perception, balance, and vestibular control that might affect several aspects of motor function, mood, and cognitive impairment of PD patients (25). Although the neuroprotective effects of dance in PD have not been adequately examined, it has been proposed that BDNF upregulation and other molecular pathways may underlie the dance-mediated enhancement of neuronal activation in disrupted sensory-motor areas in PD, thereby resulting in the improvement of motor symptoms (25).

Resistance training (RT) is a renowned part of disease-prevention and disease-therapy protocols (27). It averts muscle loss, as muscle can increase its size through hypertrophy at any age, and improves muscular strength and gait components (2, 28, 29). Muscular weakness is a resultant of PD, as inhibition activation of motor neurons leads to muscle mass losses (7). Gait disturbances, poor balance, falls, and bradykinesia also seem to be associated with lack of strength, muscular imbalances, and differences between left and right sides (2, 30).

Indeed, a review with 401 participants examining the effects of progressive RT on physical function and balance in people with PD demonstrated that after 10 weeks of such training (2–3 times per week at moderate intensity) significantly improved strength, balance, and motor symptoms (28). Other studies found that RT should be combined with different forms of training in order to improve parameters such as balance or gait (2, 29), while there was also evidence that RT improves lower limb strength but not gait and balance (31). It should be stressed that research on RT in relation to PD is rather limited with different characteristics and methodological heterogeneity such as study design, randomization, and/or measurements (2).

Previous systematic reviews have individually examined rhythmic cueing, dance, or RT in relation to PD symptomatology. However, it is not yet entirely clear with which of these three methods would provide the most benefits for different clinical aspects of PD. Therefore, the aim of the present systematic review and meta-analysis was to synthesize evidence associated with the functional and clinical effectiveness of rhythmic cueing, dance, or RT on motor and non-motor parameters in patients with PD. It is anticipated that the findings would form the basis for a new protocol synthesis aiming at improving PD symptoms, through the development of more holistic exercise interventions.

## Methodology

The present work was conducted according to the Preferred Reporting Items for Systematic Review and Meta-analysis (PRISMA) guidelines. It was registered with the International Prospective Register of systematic reviews (PROSPERO) (registration number: CRD42020212380).

### Eligibility criteria

We considered the studies of any methodological design, which included experimental groups attended any form of rhythmic cueing intervention, any type of dance, or any form of RT, in PD patients. There were no restrictions regarding the duration of interventions. Key outcome domains were gait velocity/speed, stride length, stride time, strength of lower limbs, motor symptoms, functional parameters, QoL, cognition, state of mood, and sleep disorders. Eligible control situation considered either an appropriate control group (non-active or usual care for PD) or baseline measurements that were comparable with post-intervention measurements. Animal studies, case reports, reviews, editorials, conferences, and magazine papers were excluded.

Eligibility criteria for participants were Hoehn & Yahr (H&Y) PD rating scale I–IV (32). We applied no restrictions on disease duration, age, gender, and type of drug therapy, except for stable antiparkinsonian medication. Patients with other neurological problems or deep brain stimulation, cancer, cardiovascular disease, poor visual or auditory capability, and musculoskeletal problems were excluded.

### Search and selection strategy

PubMed, Medline, Cochrane Library (trials), and SPORTDiscus were searched from the date of their inception until 1 June 2021. The key words (algorithm) used can be found in the supplement (33). The article selection was undertaken by two researchers (CK and MB). Any discrepancies have been resolved through discussion by a third researcher acting as referee (PCD). In the first step of the selection process, retrieved articles that were obviously irrelevant to our research question were excluded based on screening of titles and abstracts. Considering the aim of the current systematic review, we then checked the full texts of the remaining publications in order to select the eligible ones. Both steps were based on our inclusion and exclusion criteria.

### Data extraction

CK and MB extracted the data from the eligible studies. One referee (PCD) ensured that all the necessary data are listed in tables. These included: (1) First author name and date of publication for identification, (2) Methodological design of each study, (3) Population characteristics sample size, groups, age, gender (if available), and H&Y PD rating scale (32), (4) Intervention (type, duration, and frequency), and (5) Eligible outcomes. Outcomes were continuously presented in mean and standard deviation (SD) of unified PD rating scale part III (UPDRS-III), Timed up and go test (TUG), ten meters walk test (TMWT), gait (velocity/speed), stride length, stride duration, PD questionnaire (PDQ39) score, strength of lower limbs, Montreal cognitive assessment (MoCa), sleep disorders (PSQI), and Brunel mood state (BRUMS). Fill the above outcomes were considered as “critical and meaningful,” according to 2022 Cochrane handbook for systematic reviews (34). Included outcomes encompassed the most frequent motor and non-motor parameters that affect every-day life of people with PD (35, 36). It is noteworthy that PDQ-39 is a valid questionnaire to assess quality of life in PD (37), UPDRS-III is an effective scale to assess motor symptoms in PD (38), whereas TUG is a common test to measure functional mobility in PD (39, 40). Similarly, MoCa is a widely used test to detect even mild cognitive impairments in patients with PD (41, 42). The BRUMS (43) evaluates 6 mood states (tension, depression, anger, vigor, fatigue, and mental confusion) in different populations, including PD patients and elderly people (44–47). The extracted data used for the meta-analyses can be found in the supplement in an open depository (33).

### Risk of bias

The evaluation of the methodological quality of the eligible studies was independently completed by two researchers (CK and MB). Any conflicts arose between the two researchers, assessment and evaluation, were resolved by the referee researcher (PCD) via discussion. The ROBINS-I tool was used for non-randomized controlled trials (48), and the updated Risk of Bias 2 (ROB2) tool of Cochrane Library used for randomized controlled trials (RCT) (49).

### Data synthesis and prospective meta-analysis

For seven eligible studies (50–56), a narrative data synthesis was conducted due to unsuitable data for a meta-analysis, as means and standard deviations (SD) were not included, and we were not able to retrieve the data from the corresponding authors. In addition, two studies (52, 53) were included in the narrative data synthesis due to non-parametric data reported. It has been advised that non-parametric and parametric data should not be mixed in a meta-analysis (34). Finally, a further study (57) provided data for sleep disorders, but this entry appeared only once in the outcomes, and, as such, no meta-analysis could be conducted (34).

For the eligible publications with data suitable for a meta-analysis, a continuous random effect model was employed, with means and SD, to assess motor and non-motor symptoms between experimental and control groups or baseline and post measurements. For the motor and non-motor events, a dichotomous inverse variance random effect model meta-analysis (i.e., odds ratio) was used to assess the effects (acute or chronic) of rhythmic cueing, dance, and/or RT interventions, in patients with PD, against the incidence of an adverse effect or positive effect in a group of patients not exposed to the aforementioned interventions. For all meta-analyses, the RevMan 5.4.13 software was used (58). Outcomes in four eligible studies (53, 59–61) were reported in figures, and therefore, the WebPlotDigitizer (62) software was used to extract data for the meta-analysis. For the eligible studies (59, 63–65) with reported outcomes as means and standard errors, conversions into standard deviations were achieved using the following equation: Standard deviation = standard error${}^{*}\sqrt{n}$ (34). The 95% confidence interval and heterogeneity between the eligible studies were evaluated using the I^2^ statistic. A statistically significant result for heterogeneity was considered when *p* < 0.10, while interpretation of I^2^ index was based on the Cochrane Library Handbook (34). Finally, the standardized mean difference (SMD) was used in cases where meta-analysis included studies that assessed the same outcome but used different measurement scales. Publication bias was assessed using funnel plots, but only for those meta-analyses that include >10 studies/entries (34).

In the comparisons of group of different dance styles (64) or rhythmic cueing (66, 67), pre measurements data were considered as a control situation, and post measurements data were considered as an experimental situation. For the eligible studies (59, 60, 63, 68–72) that compared interventions of dance or RT with other activities, only dance or resistance group was considered. Control groups receiving usual care treatment were considered as appropriate, unless physical activity was part of their usual care treatment. In the absence of appropriate control group (active or healthy) (73–83) or control group (84–86), comparisons focused on pre and post measurements of experimental groups. In one study (78) that comparisons focused on less affected and most affected leg, the latter was considered. In the context of gait measurements, self-selected speed or preferred rhythm (79, 87) was chosen since these two parameters are closer to normality.

Finally, we conducted subgroup analyses to compare each one of the outcomes among rhythmic cueing, dance, and RT. In particular, gait velocity, stride length, functional mobility-TUG, Qol-PDQ-39, and motor symptoms UPDRS-III have been analyzed.

### Confidence in cumulative evidence

Meta-analyses quality of evidence was judged via the Grading of Recommendations Assessment, Development and Evaluation (GRADE) analysis (34, 88). Following previous guidelines (34, 88), we considered as an optimal information size more than 110 participants for each meta-analysis. This was based on a power analysis of a conventional sample size using three single trials (59, 66, 89).

## Results

**Figure 1:**
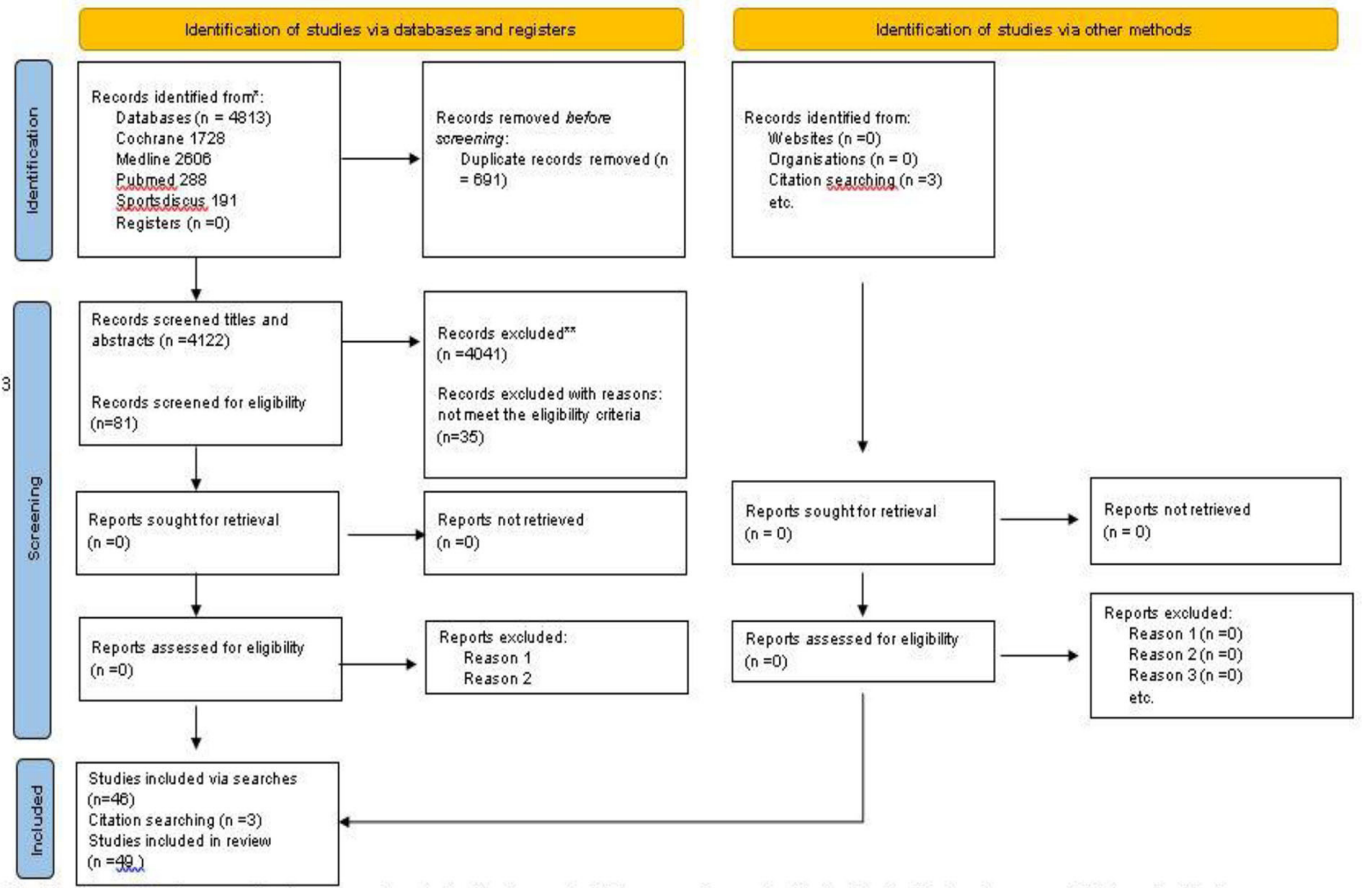
PRISMA 2020 flow diagram.

Prisma flow diagram shows information regarding article selection and characteristics of included studies (Figure 1).

We included publications from 1996 to 2021 which involved 3,767 participants (933 for rhythmic cueing, 1,470 for dance, and 1,364 for RT). Eight RCTs, two CTs, and one cohort study examined the effect of rhythmic cueing (rhythmical sounds, metronome, rhythmic styles) on PD. 10 RCTs and 12 CTs examined the effect of western theatrical (ballet, contemporary, jazz) social (Waltz, Foxtrot, Tango, Salsa, Samba, Forro), and Folklore (Irish, Sardinian, and Turo) dance protocols on PD. Twelve RCTs and four CTs studies examined the effect of RT protocols on PD. Interventions ranged from one session for a period of 24 months. The characteristics of the eligible studies are available in the supplement (Supplementary Table S1, pages 5–34) in an open depository (33).

### Search and selection outcomes

Of the 4,813 retrieved publications, 691 were duplicates and 4,039 were excluded. Of the remaining 134 publications, 53 were reviews and conference papers and 35 did not fulfill the inclusion criteria. Finally, 46 studies were classified eligible, while three additional eligible studies were found in their reference lists. The total number of eligible studies included in the systematic review was 49.

### Risk of bias assessments

Regarding the eligible RCTs, one study displayed high risk of bias (90), 12 were found with some concerns (57, 63, 65, 67–69, 73, 74, 91–94), and 18 studies displayed low risk of bias (53, 55, 60, 61, 64, 66, 70, 72, 75, 79, 81, 82, 89, 95–98), in randomization process. With respect to intervention assignment, two studies showed high risk of bias (64, 90), five studies exhibited some concerns (72, 75, 92, 95, 97), while the remaining studies disclosed low risk of bias (53, 55, 57, 60, 61, 63, 66–70, 73, 74, 79, 81, 82, 89, 91, 93, 94, 96, 98). In relation to intervention adherence, six studies displayed high risk of bias [55, 64, 72, 74, 90, 91[, eight exhibited some concerns (63, 65, 70, 81, 92, 95, 97), and 17 low risk of bias (53, 57, 60, 61, 66–69, 73, 75, 79, 82, 89, 93, 94, 96, 98). Considering missing data, three studies displayed some concerns (53, 57, 72), while the remaining studies showed low risk of bias (55, 60, 61, 63–70, 73–75, 79, 81, 82, 89–98). In relation to bias outcome, five studies exhibited some concerns (72–74, 81, 96), and the remaining studies presented low risk of bias (55, 60, 61, 63–70, 72, 75, 79, 82, 89–95, 97, 98). In bias reported outcomes, two studies presented high risk of bias (60, 90) one study displayed some concerns (81) and 27 studies revealed low risk of bias (53, 55, 57, 61, 63–70, 72–75, 79, 82, 89, 91–98).

Regarding the eligible CTs, two studies displayed moderate risk of bias (51, 80) and the remaining studies low risk (31, 50, 52, 54, 56, 59, 76–78, 83–87, 99–101). For bias selection, one study displayed serious risk of bias (99), nine studies showed moderate risk of bias (50, 51, 54, 59, 76, 77, 83, 87, 101), and nine studies showed low risk of bias (31, 52, 56, 78, 80, 83, 84, 86, 100). Regarding bias classification, seven studies showed moderate risk (23, 50, 76, 78, 83, 85, 99) and 12 studies low risk in bias (31, 51, 52, 54, 56, 59, 77, 80, 84, 86, 87, 100). For association to bias deviation of intervention, all studies (23, 31, 50–52, 54, 56, 59, 76–78, 80, 83–87, 99, 100) displayed low risk. Three studies displayed moderate risk (80, 85, 100), and 16 studies low risk in bias missing data (23, 31, 50–52, 54, 56, 59, 76–78, 83, 84, 86, 87, 99). For bias outcome, one study displayed some concerns (76), 14 studies moderate (23, 31, 50, 51, 54, 56, 76–78, 80, 83, 85, 87, 99), and five studies (51, 52, 84, 86, 100) displayed low risk. In bias reported results, three studies showed moderate risk (50, 56, 85) and 16 (23, 31, 51, 52, 54, 59, 76–78, 80, 83, 84, 86, 87, 99, 100) displayed low risk of bias. Risk of bias outcomes can be found in Supplementary Tables S2, S3 and Supplementary Figures S1, S2 (33).

### Narrative data synthesis

In relation to the effects of rhythmic cueing on PD, one study examined the acute effects of rhythmic auditory stimulation (RAS) on gait velocity, indicating that RAS can facilitate locomotion (50). Similarly, another study (54) reported that rhythmic auditory cues significantly increased gait parameters, such as walking velocity and stride length, after 8 weeks of training. However, the use of metronomes did not improve mobility or physical functioning or other aspects of QoL (55).

In relation to the effects of dance, one study revealed that Irish dance may improve QoL (52), but another set of data (53) revealed that Irish dance does not improve QoL. A 12-month classical ballet did not affect gait variability (51), but an 8-month dance for PD did improve functional mobility and QoL in patients with PD (56). With respect to RT, a 12-week progressive RT improved sleep quality in this population (57).

However, the narrative review included a small number of studies, and therefore, it is difficult to evaluate the relevance of the findings.

### Meta-analysis outcomes

In the supplement (S) of the following can be found: (a) forest plots of rhythmic cueing (Supplementary Figures S3A–C, S6A,B), (b) forest plots of dance (Supplementary Figures S4A–C, S7A–C), (c) funnel plots of dance 4Ba and 4Ca, and d) RT (Supplementary Figures S5A–D, S8A–D). The data used for the meta-analyses can be found in an open depository (33).

#### Rhythmic cueing

Meta-analysis results revealed significant effects of rhythmic cueing on gait velocity [(SMD = 0.54, CI = 0.21–0.88, Z = 3.20, *I*^2^ = 46%, *p* = 0.01, (Supplementary Figure S3A)] and stride length [MD = 0.09, CI = 0.03–0.15, Z = 3.08, *I*^2^ = 37%, *p* = 0.01, (Supplementary Figure S3B)], whereas no significant effects have been observed on stride time [SMD = 0.21, CI = −0.57 to 0.14, Z = 1.17, *I*^2^ = 0%, *p* = 0.20, (Supplementary Figure S6A)] in PD patients. Furthermore, rhythmic cues significantly improved motor symptoms-UPDRS-III [MD = −3.94, CI = (−7.47) – (−0.41), Z = 2.19, *I*^2^ = 7%, *p* = 0.03, (Supplementary Figure S3C)]. No effects of rhythmic cueing have been observed on functional mobility-TUG [MD = 2.31, CI = −7.83, 3.21, Z = 0.82, *I*^2^ = 75%, *p* = 0.41, (Supplementary Figure S6B)].

#### Dance

Dance interventions for PD significantly improved stride length [MD = 0.07, CI = 0–0.15, Z = 1.97, *I*^2^ = 0%, *p* = 0.05, (Supplementary Figure S4A], functional mobility-TUG [MD = −1.26, CI = (−1.77) -(−0.75), Z = 4.82, *I*^2^ = 0% *p* = 0.01, (Supplementary Figure S4B)], and motor symptoms-UPDRS-III [MD = −5.38, CI = (−8.44) – (−2.32), Z = 3.44, *I*^2^ = 79%, *p* = .01, (Supplementary Figure S4C)]. On the contrary, no significant effects have been observed on gait velocity [SMD = 0.19, CI = −0.06, 0.44, Z = 1.52, *I*^2^ = 0%, *p* = .13 (Supplementary Figure S7A)], quality of life-PDQ39 [MD = −2.19, CI = −6.21, 1.84, Z = 1.07, *I*^2^ = 34%, *p* = 0.29 (Supplementary Figure S7B)], and cognition-MoCa [MD = 0.60, CI = −0.78, 1.97, Z = 0.85, *I*^2^ = 0%, *p* = 0.13 (Supplementary Figure S7C)].

#### Resistance training

Significant positive effects of RT in PD have been observed on functional mobility-TUG [MD = −1.75, CI = (−3.07)-(−0.44), Z = 2.61, *I*^2^ = 81%, *p* = 0.01, (Supplementary Figure S5A)], quality of life-PDQ-39 [SMD = 0.38, CI = (−0.67)–(−0.09), Z = 2.58, *I*^2^ = 31%, *p* = 0.01, (Supplementary Figure S5B)], leg press [SMD = 3.51, CI = 1.50–5.52, Z = 3.42, I^2^ = 91%, *p* = 0.01, (Supplementary Figure S5C)], and knee flexion [SMD = 1.00, CI = 0.18–1.82, Z = 2.40, *I*^2^ = 65%, *p* = 0.02, (Supplementary Figure S5D)]. No significant effects have been found on gait velocity/speed [SMD = 0.32, CI = −0.13, 0.77, Z = 1.37, I^2^ = 50%, *p* = 0.17, (Supplementary Figure S8A)], stride length [MD = 0.05, CI = −0.05, 0.16, Z = 1.96, *I*^2^ = 0%, *p* = 0.34, (Supplementary Figure S8B)], in motor symptoms-UPDRS-III [MD = −2.74 CI = −5.55, 0.07, Z = 1.91, *I*^2^ = 1%, *p* = 0.06, (Supplementary Figure S3B)], and knee extension [SMD = 0.88, CI = −0.54, 2.30, Z = 1.22, *I*^2^ = 91%, *p* = 0.22, (Supplementary Figure S8D)].

#### Subgroup analyses of the outcomes between rhythmic cueing, dance, and resistance training

**Figure 2:**
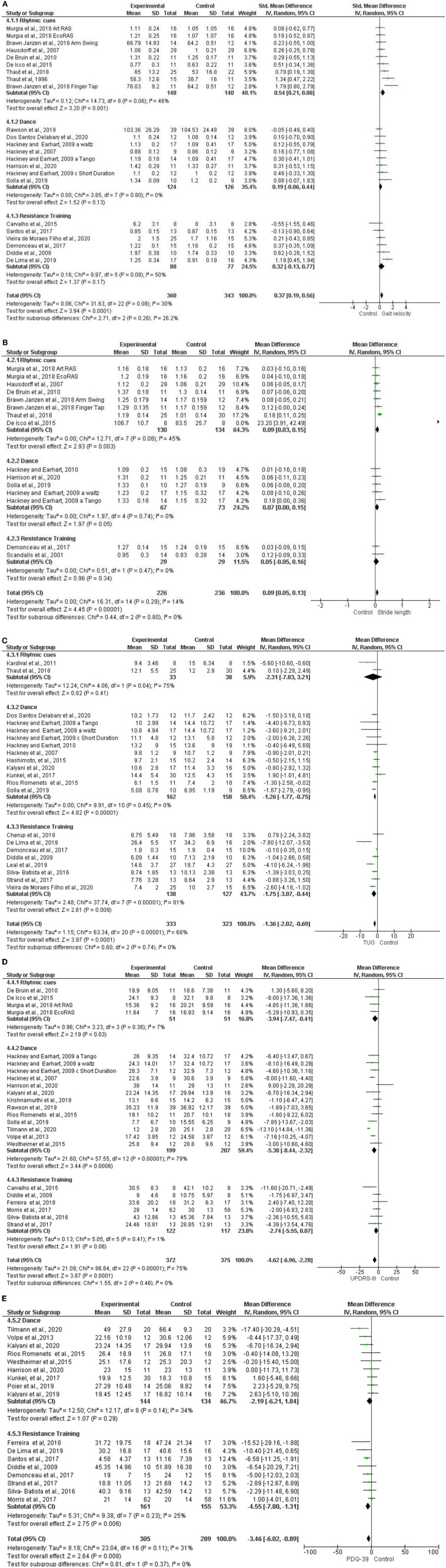
**(A)** Subgroup analysis of rhythmic cueing, dance and RT of Gait Velocity. **(B)** Subgroup analysis of rhythmic cueing, dance, and RT of stride length. **(C)** Subgroup analysis of rhythmic cueing, dance, and RT of functional mobility - TUG. **(D)** Subgroup analysis of rhythmic cueing, dance, and RT of motor symptoms - UPDRS-III. **(E)** Subgroup analysis of dance and RT of QoL-PDQ-39.

Subgroup analyses have shown non-significant differences between groups (Rhythmic cueing, Dance, RT) in gait velocity [SMD = 0.37, CI = 0.19, 0.56, *I*^2^ = 26.2%, *p* = 0.26, Figure 2A], while we found a significant overall effect [Z = 3.94, *p* < 0.0001]. Non-significant differences between groups have been observed (Rhythmic cueing, Dance, RT) in stride length [SMD = 0.09, CI = 0.05, 0.13, *I*^2^ = 0 %, *p* = 0.80, Figure 2B], while we observed a significant overall effect [Z = 4.45, *p* < 0.00001]. Similarly, non-significant differences have been found between groups (Rhythmic cueing, Dance, RT) in functional mobility-TUG [MD = −1.36, CI = (−2.02, −0.69), *I*^2^ = 0%, *p* = 0.74, Figure 2C], but a significant overall effect [Z = 3.97, *p* < 0.0001]. Non-significant differences between groups have been revealed in motor symptoms-UPDRS-III [MD = −4.62, CI = (−6.96, −2.28), *I*^2^ = 0%, *p* = 0.46], while we detected a significant overall effect [Z = 3.87, *p* < 0.0001, Figure 2D]. Non-significant subgroup (Dance, RT) differences have further been observed for quality of life-PDQ-39 [MD = −36, CI = (−6.02, −0,89), *I*^2^ = 0%, Figure 2E], coupled with a significant overall effect [Z = 2.64, *p* < 0.008].

#### Confidence in cumulative evidence outcomes

GRADE analysis outcomes can be found in the supplement (Supplementary Table S4) in an open depository (33). The meta-analyses of the effects of rhythmic cueing on gait velocity (#1) and stride length (#2) displayed moderate quality, while on stride time (#3), the quality was very low. The meta analysis of the effects of rhythmic cueing on functional mobility TUG (#4) and motor symptoms-UPDRS-III (#5) displayed low quality. The meta-analyses of the effect of dance on gait velocity (#6), stride length (#7), and functional mobility-TUG (#8) exhibited moderate quality. The meta-analyses for motor symptoms-UPDRS-III (#10) exhibited very low quality, whereas QoL-PDQ39 (#10) and cognition-MoCa (#11) exhibited moderate quality. The meta-analyses focused on the effects of RT displayed moderate quality for gait velocity (#12) and very low for stride length (#13); yet, low quality for functional mobility-TUG (#14) and moderate for motor symptoms-UPDRS-III (#15). The meta-analyses of the effects of RT displayed moderate quality for QoL-PDQ39 (#16), low for leg press (#17), very low for knee flexion (#18), and low quality for knee extension (#19).

## Discussion

The aim of the present systematic review and meta-analysis was to synthesize evidence associated with the functional and clinical effectiveness of rhythmic cueing, dance, or RT on motor and non-motor parameters in patients with PD. We found that the aforementioned forms of exercise positively affect the examined outcomes, with rhythmic cueing to be associated with three outcomes (Gait, Stride, and UPDRS-III), dance with three (TUG, Stride, and UPDRS-III), and RT with two outcomes (TUG and PDQ-39). However, there is no sufficient evidence to recommend which of these interventions has the greatest effects.

### Completeness of evidence

#### Rhythmic cueing

There was sufficient evidence to assess the effects of rhythmic cueing on gait velocity (nine included in meta-analysis/nine eligible) and stride length (nine included in meta-analysis/nine eligible). The sample was of optimal information size (>110), and GRADE analysis displayed moderate quality of evidence, indicating that rhythmic cueing could be treated as an effective intervention for improving gait characteristics (12, 17). Similarly, there was sufficient evidence to assess the effects of rhythmic cueing on motor symptoms-UPDRS-III (four included in meta-analysis/nine eligible), but the sample size was relatively small (<110), and GRADE analysis displayed low quality of evidence.

#### Dance

There was sufficient evidence (>110 participants) assessing the effects of dance protocols on functional mobility-TUG (11 included in meta-analysis/19 eligible), motor symptoms-UPDRS-III (13 included in meta-analysis/19 eligible), and stride length (five included in meta-analysis/19 eligible) in patients with PD. Although GRADE analysis revealed moderate quality for functional mobility-TUG, very low for motor symptoms-UPDRS-III, and moderate quality for stride length, findings indicate the efficacy of dance for improving mobility in this population (22, 102).

#### Resistance training (RT)

There was sufficient evidence assessing the effects of RT on QoL-PDQ39 (eight included in meta-analysis/16 included studies) with a sample size of >110 and functional mobility-TUG (eight included in meta-analysis/16 eligible) with a sample size of >110. Although GRADE analysis displayed moderate for QoL-PDQ39 and low quality for functional mobility-TUG, it could be argued that RT seems to regulate the majority of parameters associated with daily life. Also, there was sufficient evidence for leg press (four included in meta-analysis/16 eligible) with a sample size of >110, and to a lesser extent for knee flexion (three included in meta-analysis/16 eligible) with a sample size of <110. The aforementioned findings suggest that RT may activate cellular adaptive mechanisms thus, improving muscle strength (2, 103).

### Subgroup analysis of the outcomes for rhythmic cueing, dance, and resistance training

Gait velocity, stride length, functional mobility-TUG, motor symptoms UPDRS-III, and Qol-PDQ-3 outcomes were analyzed. Stride time outcome has been detected in rhythm group only, and therefore, was excluded from the subgroup analysis. Also, cognition-MoCa was excluded from the subgroup analysis as it was only detected in the dance group. Similarly, knee flexion, knee extension, and leg press outcomes were detected in RT group only, and they were not included in the subgroup analyses.

### Comparative perspective and applicability of evidence

Subgroup analyses have shown that all three forms of exercise are effective in patients with PD, supporting our hypothesis referring to a holistic approach. This stems from the fact that only outcome common to all three forms of exercise were incorporated in these analyses (Figures 2A–E).

Furthermore, meta-analyses have shown that rhythm cueing improves gait parameters, such as gait velocity, stride length, and motor symptoms, whereas dance seems to improve stride length, motor symptoms, and functional mobility. RT helps to improve QoL, functional mobility and, at the same time, enhances muscular strength in lower limbs. These findings support the notion that a protocol combining rhythmic cues, dance, and RT would probably provide a more holistic approach for improving PD manifestation.

We may theorize that the non-significant effects of dance on QoL could be attributed to the fact that dance is a complicated activity (104), especially for people who experience cognitive impairment in attention, visuospatial skills, and memory. For instance, Western theatrical dance or social dances are complicated activities containing movement combinations, whereas each class may include sections such as rhythm part, improvization, mime, and choreographies. Given that PD symptoms vary from person to person with some patients experiencing cognitive decline, the perception and understanding of movements in a dance class may be stressful for some patients. Relatively, on the one hand, recent systematic reviews examining the impact of dance on QoL revealed contradictory results suggesting that further research is needed (22, 104). On the other hand, a 2021 systematic review provided positive evidence on the effect of dance on quality of life, but the sample size was rather small and prevented generalization (105). An explanation for the aforementioned results may be the complexity of dance activity itself, which renders existing questionnaires not sensitive enough to fully capture elements of QoL (104).

### Strengths and limitations

To the best of our knowledge, this is the first systematic review and meta-analysis on the effects of rhythmic cues, dance, or RT on PD patients. We searched appropriate databases to develop the key word algorithms, using standardized indexing terms, MeSH terms, and truncations, in order to retrieve publications relevant to our research question (34), while two independent investigators performed the searching, selection, data extraction, and risk of bias assessments.

The current narrative data synthesis included a relatively small number of studies (nine out of 50), which may impose a difficulty to merge their findings with those from the meta-analyses. We did not detect eligible articles for evaluating the state of mood - BRUMS. If more commonly used measures of mood were included in the search, then some effects of the interventions may have been found.

Other limitations include variations in methodological designs, while there was no material indicating whether protocols were designed according to participants' symptomatology. Also, eligible studies did not differentiate disease stages. None of the eligible studies examined fatigue factors, and we detected no information regarding the intensities of dance interventions in most studies. Duration, frequency, and intensity of physical activities are crucial, as fatigue may be an inhibitory factor in parkinsonian populations, similar to that in athletic populations (106, 107). Finally, the eligible dance studies included different dance genres with little information on the structure and/or content.

## Conclusions

The present systematic review and meta-analysis indicates that rhythmic cues, dance, or RT positively affect the examined outcomes, with rhythmic cueing to be associated with three outcomes (Gait, Stride, and UPDRS-III), dance with three (TUG, Stride, and UPDRS-III), and RT with two outcomes (TUG and PDQ-39). Subgroup analyses confirmed the beneficial effects of these forms of exercise. Clinicians should entertain the idea of more holistic exercise protocols aiming at improving PD manifestations. Future studies should consider (a) implementation of exercise protocols based on PD patients' symptomatology and disease duration, and (b) standardization of test protocols.

## Data availability statement

The datasets presented in this study can be found in online repositories. The names of the repository/repositories and accession number(s) can be found in the article/Supplementary material.

## Author contributions

CK and YK did the conceptualization. CK and PD designed the algorithm. CK and MB did article selection and Risk of Bias and the evaluation of the methodological quality of the eligible studies was independently completed. PD acted as referee. CK did the statistical analysis, edited tables and pictures, and wrote the primary manuscript. AH contributed to the methodology and the article. MW, SP, and EA contributed to the article. CK, PD, and YK reviewed the final manuscript. All authors approved the submitted version.

## Conflict of interest

The authors declare that the research was conducted in the absence of any commercial or financial relationships that could be construed as a potential conflict of interest.

## Publisher's note

All claims expressed in this article are solely those of the authors and do not necessarily represent those of their affiliated organizations, or those of the publisher, the editors and the reviewers. Any product that may be evaluated in this article, or claim that may be made by its manufacturer, is not guaranteed or endorsed by the publisher.
