# Supplementary material for: Rhythmic cueing, dance, resistance training, and Parkinson's disease: A systematic review and meta-analysis
Source: Front Neurol. 2022 Aug 9;13:875178. doi: 10.3389/fneur.2022.875178 (PMC9413961; doi:10.3389/fneur.2022.875178)
Supplement: Supplementary file 1 [file Data_Sheet_1.pdf]

Supplementary Material

Rhythmic cueing, Dance, Resistance Training and Parkinson disease: A systematic Review and Meta-Analysis

Claire Chrysanthi Karpodini,[1] Petros C. Dinas,[2] Efthalia Angelopoulou[3], Matthew A. Wyon,[1] Aline Nogueira Hass,[4] Maria Bougiesi,[2] Sokratis G. Papageorgiou[3], Yiannis Koutedakis[1,2]

[1]Sport and Physical Activity Research Centre, Faculty of Education, Health and Wellbeing, University of Wolverhampton, Walsall, United Kingdom, [2]FAME Laboratory, Department of Physical Education and Sport Science, University of Thessaly, Greece
[3]1st Department of Neurology, Medical School, National and Kapodistrian University of Athens, Eginition University Hospital, Athens, Greece.
[4]Federal University of Rio Grande do Sul, Brazil (UFRGS), School of Physical Education Physiotherapy and Dance, Brazil

*** Correspondence:**
Email: C.C.Karpodini2@wlv.ac.uk
claire_karpodini@outlook.com

Contents

Key word algorithm 4

Table 1: Data extraction, characteristics of eligible studies Group 1 Rhythm, Group 2 Dance, Group 3 Strength (RT) 5

Table 2: Randomized control trials (RCTs) Risk of bias assessment outcomes........ 33

Key: **+** : low, **?** : some concerns, **-** : high, **N**: not applicable. 33

Figure 1: RCTs Risk of bias assessment outcomes 34

Table 3: Clinical Trials (CTs) Risk of bias assessment outcomes Key: ++: low, +: moderate, **?** : some concerns, **-** :serious, **--** :critical, **---** : high **NI** : no information. 35

Figure 2: CTs Risk of bias assessment outcomes 36

Figure 3: Forest plots of significant effects of rhythmic cueing on PD 37

- **3A:** Forest plot of significant effects of rhythmic cueing on gait velocity........ 37
- **3B:** Forest plot of significant effects of rhythmic cueing on stride length........ 38
- **3C** Forest plot of significant effects of rhythmic cueing on motor symptoms-UPDRS-III 39

Figure 4: Forest plots of significant effects of dance on Parkinson disease........ 39

- **4A**: Forest plot of significant effects of dance on stride length........ 39
- **4B**: Forest plot of significant effects of dance on functional mobility-TUG........ 40
- **4Ba**: Funnel Plot of significant effects of dance on functional mobility-TUG 41
- **4C**: Forest plot of significant effects of dance on motor symptoms-UPDRS-III 42
- **4Ca**: Funnel plot of significant effects of dance on motor symptoms-UPDRS-III 43

Figure 5: Forest plots of significant effects of RT on PD 44

- **5A**: Forest plot of significant effects of RT on functional mobility-TUG 44
- **5B**: Forest plot of significant effects of RT on QoL- PDQ-39 45
- **5C**: Forest plot of significant effects of RT on lower limb strength- Leg Press........ 45
- **5D**: Forest plot of significant effects of RT on lower limb strength- knee flexion 46

Figure 6: Forest plot of non-significant effects of rhythmic cueing on PD........ 46

- **6A:** Forest plot of non-significant effects of rhythmic cueing on stride time 46
- **6B** Forest plot of non-significant effects of rhythmic cueing on functional mobility-TUG 47

Figure 7: Forest plots non-significant effects of dance on PD 47

- **7A:** Forest plot of non-significant effects of dance on gait velocity 47
- **7B:** Forest plot of non-significant effects of dance on QoL-PDQ-39 48
- **7C**: Forest plot of non-significant effects of dance on cognition-MoCa........ 49

Figure 8 Forest plots non-significant effects of resistance training (RT) on Parkinson Disease 49

- **8A**: Forest plot of non-significant effects of RT on gait velocity........ 49
- **8B**: Forest plot of non-significant effects of RT on stride length........ 50

8C: Forest plot of non-significant effects of RT on motor symptoms-UPDRS-III 50
8D: Forest plot of non-significant effects of RT on lower limb strength-knee extension ... 51
Table 4: GRADE analysis outcomes 52
PRISMA **CHECKLIST** 63
PRISMA 2020 Main Checklist 63
PRIMSA Abstract Checklist 66

Key word algorithm

(((Parkinson disease[Title/Abstract] OR Parkinsonian[Title/Abstract]) AND (Rhythmic movement[Title/Abstract] OR movement[Title/Abstract] OR Motor activity[Title/Abstract] OR Folklore[Title/Abstract] OR Tango[Title/Abstract] OR Samba[Title/Abstract] OR Dance*[Title/Abstract] OR Theatre dance[Title/Abstract] OR Musical[Title/Abstract] OR Ballet[Title/Abstract] OR Contemporary Dance[Title/Abstract] OR Strength training[Title/Abstract] OR Resistance training[Title/Abstract]) AND (Gait[Title/Abstract] OR Functional mobility[Title/Abstract] OR Strength of lower limbs[Title/Abstract] OR MDS-UPDRS[Title/Abstract] OR H & Y scale[Title/Abstract] OR Quality of life[Title/Abstract] OR PDQ39[Title/Abstract] OR Cognitive performance[Title/Abstract] OR MoCa[Title/Abstract] OR State of mood[Title/Abstract] OR BRUMS[Title/Abstract] OR Sleep disorders[Title/Abstract] OR Rhythmic perception[Title/Abstract] OR Rhythmic cues[Title/Abstract] OR Fitness level[Title/Abstract])) NOT ((animals[MeSH Terms]) NOT (humans[MeSH Terms])))

Table 1: Data extraction, characteristics of eligible studies Group 1 Rhythm, Group 2 Dance, Group 3 Strength (RT)

Group	First Author, Date	Study Design	Participants	Methods	Main Findings
Group 1 Rhythm	Thaut *et al.*, 1996	RCT	EX: n= 15 (10 men; 69 ±8 years; H&Y scale: 2.4).<br>SPT: n=11 (8 men; 74±3 years; H&Y scale: 2.5).<br>NT: n= 11 (8 men; 71±8 years, H&Y scale:2.6).	**Duration:** 3 weeks, 30 min daily.<br>EG=gait training with RAS<br>SPT=gait training without RAS.<br>NT= follow their normal activities.<br>**Time Points:**<br>Pre- test<br>Post-test.	**M and SD**<br>RAS training improved gait velocity and stride length significantly after<br>only 3 weeks.<br>**Post- test-week 3**<br>EX<br>Flat Velocity (m/min): 58.3±12.6<br>Stride (m): 1.10±0.17<br>NT<br>Flat Velocity (m/min): 38.7±16.<br>Stride (m): 1.01±0.14
Group 1 Rhythm	McIntosh *et al.,* 1997	Cohort	ON: n= 21(15 men; 67-75 years).<br>OFF: n= 10 (6 men; 70-76 years).<br>H&Y scale:2-4.<br>C: n= 10 healthy (4 men; 67-77 years).	**Duration:** One session.<br>All groups walking distance of 30m.<br>**Time points** as follows:<br>Baseline.<br>Their own max speed with no external rhythm.<br>In time to RAS in tempo to each patient's, baseline cadence.<br>In time to RAS, tempo 10% faster than baseline.	**M and SD**<br>The results of this study provide strong evidence that rhythmic auditory stimulation can<br>facilitate locomotor function in patients with Parkinson's disease suggesting rhythmic entrainment as a possible facilitating mechanism. A technique for gait rehabilitation in PD.

				No external rhythm (carry over effects).	
Group 1 Rhythm	Hausdorff *et al.,* 2007	CT	PD: n= 29 (67.2 ± 9.1 years, H&Y scale: 2-3).<br>CTRL: n=26 (healthy 64.6 ±6.8 years).	**Duration:** One session.<br>All groups: The effects of RAS on gait, in 100m walking distance.<br>**Time points** as follows:<br>baseline (comfortable pace without RAS),<br>RAS= 100% of the baseline step rate<br>RAS= 110% of the baseline step rate.<br>**immediate carryover effects(ICE): without RAS**<br>delayed carryover effects (DCE): without RAS after a 15-min rest.	**M and SD**<br>**PD**<br>RAS enables more automatic movement and reduces stride-to-stride variability in patients with PD.<br>**Baseline**<br>Gait speed (m/s): 1.00 ± 0.21<br>Stride length(m): 1.06 ± 0.21<br>**Post Measurements Immediate carry over effects of PD at 100%**<br>Gait speed(m/s): 1.06 ± 0.24<br>Stride length(m):1.10 ± 0.23
Group 1 Rhythm	Ford et al., 2010<br>Narrative	Clinical Trial	PD: n= 12 (50–79yr; 7 men)<br>H&Y scale 1-3	Duration: 8 weeks 30min, 3 /week.<br>Training<br>started at an external auditory cue rate (available in 5-beat increments from 60–165bpm) that approximated the comfortable cadence that was previously measured in the laboratory.<br>Time points<br>Pre-Intervention Post- Intervention	**Means**<br>Significant increase in walking velocity, stride length, and cadence after 8 weeks of<br>training.<br>**Comfortable pace**<br>**Baseline**<br>Gait speed (m/s): 0.99<br>Stride length(m): 1.13<br>**Post**<br>Gait speed(m/s): 1.17<br>Stride length(m): 1.26
Group 1 Rhythm	Elston J *et al.,* 2010)	RCT	EARLY: n= 21 (13 men, 71.5 ± 11.3 years).	**Duration:** 14 weeks, Daily.<br>Daily activities and not only	**M and SD**<br>Data were in mean difference

			LATE: n=20 (15 men, 70.4 ±8.7 years).<br>H&Y scale 2-4	walking per se as follows:<br>**4 weeks:**<br>EARLY with metronome.<br>LATE without metronome.<br>**6 weeks:** wash out period (both groups without metronome).<br>**4 weeks:**<br>EARLY with no metronome.<br>LATE with metronome.<br>**Time points:**<br>Baseline- Test 1<br>Week 4 - Test 2<br>week 10 -Test 3<br>week 14- Test 4	The use of metronomes<br>without therapy for four weeks in people with moderate to severe Parkinson's disease does not dramatically improve mobility and ADL, social and physical functioning or other domains of quality of life.
Group 1 Rhythm	De Bruin et al., 2010	RCT	MUSIC: n= 11 (6 men, 64.1±4.2 years).<br>CTRL: n=11 (5 men 67.0 ±8.1 years).<br>H&Y scale 1-3	**Duration:** 13 weeks, 30 minutes, 3/week<br>MUSIC GROUP: Walking at a comfortable pace whilst listening to an individualized music playlist through head/ear-phones, in addition to maintaining regular activities.<br>CTRL GROUP: continued with any regular activities.<br>**Time points:**<br>Pre-intervention<br>Post-intervention – week 13.	**M and SD**<br>**Post measurements –week 13**<br>The MUSIC group improved<br>gait velocity, stride time, and motor symptom severity following the intervention.<br>MUSIC GROUP:<br>Gait;<br>Velocity(m/s): 1.31± 0.22<br>Stride time (s): 1.06±0.07<br>Stride length (m): 1.37±0.18<br>UPDRS -III:19.9±9.05<br>CTRL GROUP:<br>Gait;<br>Velocity(m/s): 1.25±0.17

					Stride time (s): 1.06±0.13<br>Stride length (m):1.30±0.14<br>UPDRS-III:18.6±7.38
Group 1 Rhythm	Kadivar et al.,2011	RCT	RAS: n= 8 (5 men; 64-81 years).<br>Non- RAS: n=8 (6 men; 59-78).<br>H&Y scale: 2-4	**Duration:** 6-weeks; 45-60min; 3 times/week.<br>RAS=Multidirectional Step Training with RAS at 1-3 speeds normal/comfortable<br>cadence and 10% to 20% faster and slower than normal<br>cadence according to individual preference/ability within the 10% to 20% range.<br>NON-RAS=protocol as above without RAS.<br>**Time points:**<br>Pre-test, on day 1 of training (PRE)<br>Post-test, on the last day of training week 6 (POST).<br>Follow-up;<br>1 week (POST+1),<br>4 weeks (POST+4)<br>8 weeks (POST+8)	**M and SD**<br>**PRE TEST, POST –week 6**<br>Individuals with PD can generalize motor improvements achieved during multidirectional step training to contexts of functional gait and balance. Training with<br>RAS is advantageous for enhancing functional gait improvements and the maintenance of functional gait and balance improvements over 8 weeks.<br>**Secondary Outcomes**<br>RAS<br>**Baseline**<br>TUG:15±6.35<br>**Post measurements** TUG:9.44±3.46
Group 1 Rhythm	De Icco *et al.*,2015	RCT	ACOUSTIC: n=11 (78 ± 6.1 years).<br>VISUAL: n=11 (73.2±6.9 years).<br>CTRL: n=24 (72.1±7.3 years).<br>H&Y scale 2-4	**Duration:** 4 weeks; 20min gait training; 40-min rehab exercises<br>5 times/ week<br>ACOUSTIC =3 treatments for gait training:	**M and SD.**<br>**T1 and T2.**<br>ACOUSTIC<br>All 3 types of training improved gait speed;<br>Acoustic cues reduced the number of strides and

				walking in the presence of rhythmical sounds<br>VISUAL=Walking on stripes of contrasting color with respect to the floor<br>CTRL=Over ground gait training without cues<br>**Time points:**<br>TO- baseline<br>T1- week 4<br>T2 - 3 Months Later	increased stride length. The changes were not retained at T2 in any of the experimental groups. Our findings support and characterize the usefulness of cueing strategies in the rehabilitation of gait in PD.<br>**Baseline-T0**<br>UPDRS –III 32.1 ± 9.8<br>Stride duration (ms): 1250.5 ± 317.2<br>Stride length (cm): 83.5 ± 10.725.7<br>Speed(m/s):0.63 ± 0.22<br>**Post - measurements -T1**<br>Stride duration (ms): 1246 ± 263.4<br>Stride length (cm): 106.7 ± 10.7<br>Speed(m/s):0.77 ± 0.3<br>UPDRS-III 24.1 ± 9.3
Group 1 Rhythm	Braun Janzen *et al.*, 2019	RCT	FINGER TAPPING: n=11 (7 males ;68.4 ±5.31yr)<br>ARM SWING: n=14 (6 males; 64.2 ±6.32 yr)<br>CTRL: n=12 (4 males; 67.28 ±3.14 yr)<br>H&Y scale:I-II	**Duration:** One bout;<br>Rhythmic Auditory Stimulation training consisted of three blocks of 1-minute training with 30 seconds of rest in between blocks.<br>CTRL group: did not undergo any training<br><br>**Time points:**<br>Pre training<br>Post training (immediate effects)	**M and SD**<br>Rhythmic priming is possible across effector systems. Rhythmic Auditory Stimulation training of finger movements had immediate effects on gait velocity and cadence of patients with Parkinson's Disease. Swinging the arms in an alternating motion in synchrony with the metronome did not change gait speed.<br>**Post- measurements**<br>**FINGER TAPPING:**<br>Velocity(m/min): 76.03± 9.20 Stride length(m): 1.29±0.135<br>**ARM SWING**<br>Velocity(m/min): 66.79 ±14.93 Stride length(m): 1.25± 0.179

					**CTRL**<br>Velocity(m/min): 64.20 ±10.51<br>Stride length (m): 1.17 ±0.159
Group 1 Rhythm	Murgia et al.,2018	RCT	ERAS: n=19<br>(66.5±10.9 years)<br>Analyzed 16<br>ARAS: n=19 (96.9±10.1 years)<br>Analyzed 16<br>H&Y scale: 1-3	**Duration:** 5 weeks with 45 min. 2/week and 30min 3/week at home.<br>Both groups The standard and personalized exercises and 20 min specific gait<br>training with RAS, with participants engaged in walking while listening to their own personalized soundtrack (either ecological or artificial).<br>Home based program:<br>a subset of the same exercises typically<br>performed at the hospital and 30 min of gait training with RAS.<br>**Time points:**<br>TO: Baseline<br>T5: Week 5<br>T7: week 17 (3 months after the completion of the intervention)	**Mean and SD**<br>RAS is equally effective compared to artificial RAS in the management of gait disturbances associated with Parkinson's disease.<br>**Baseline-T0**<br>Stride length (m)<br>ERAS: 1.16 ± 0.20<br>ARAS: 1.13 ± 0.20<br>Gait Speed (m/s)<br>ERAS: 1.07±0.24<br>ARAS: 1.05±0.29<br>Step length (m)<br>ERAS:0.52 ± 0.12<br>ARAS: 0.52 ± 0.12<br>UPDRS III<br>ERAS: 16.93 ± 9.14<br>ARAS: 20.21 ± 9.59<br>**Post measurements**<br>**T5-end of week 5**<br>Stride length(m)<br>ERAS:1.20 ± 0.19<br>ARAS: 1.16 ± 0.18<br>Gait Speed m/s<br>ERAS: 1.21±0.25<br>ARAS: 1.11±0.24

					UPDRS-III:<br>ERAS: 11.64 ± 7.00<br>ARAS: 15.36 ± 9.20
Group 1<br>Rhythm	Thaut *et al.,*2018	RCT	EX: n=25<br>CTRL: n=22<br>(62-82 Years).<br>H&Y scale: 3-4	**Duration:** 24-weeks, 30min daily.<br>EX=home-based gait training with RAS. 2/4 tempo- 3 metronome rates:<br>- 8 weeks: 100%, 105%, 110%.<br>- 16 weeks: 105%, 110%, 115%.<br>**Time points:** Baseline<br>week 8<br>week 16<br>week 24	**M and SD**<br>**Week 24**<br>RAS training significantly reduced the number of falls in Parkinson's disease and modified<br>key gait parameters, such as velocity and stride length.<br>**EX**<br>**Baseline**<br>Velocity(m/min):<br>53±16.8.<br>Stride length(m): 1.01±0.14.<br>TUG Test (sec):12±2.9.<br>**Post measurements –week 24**<br>Velocity(m/min):<br>65±13.2. P < 0.005<br>Stride length(m): 1.19±0.14. P < 0.005<br>TUG Test (sec):12.1±5.5.
Group 2<br>Dance	Hackney *et al.,* 2007	RCT	TANGO: n=9 (6 males; 72.6± years<br>EXERCISE: n=10 (6 males; 69.6±2.1)<br>H&Y scale: 1-3	**Duration:** 13 weeks- total of 20 sessions, 60-min approximately 2/week<br>TANGO=postural stretches, balance exercises, tango-style walking, footwork<br>patterns, and experimentation with timing of steps to music, both with and without a partner.	**Means and SE (SD)**<br>Significant improvements in overall UPDRS score and non-significant improvements in self-reported Freezing of Gait. In addition, the tango group showed significant improvements<br>on the Berg Balance Scale and a trend toward improvement on the Timed Up and Go test.<br>**TANGO**<br>**Baseline**

				EXERCISE= breathing and stretching exercises, and progressed to resistance and dexterity exercises.<br>**Time points:**<br>Pre-test<br>Post-test, week 13	UPDRS-III:30.6±1.3(3.9)<br>TUG:10.7± 0.4 (1.2)<br>Gait velocity (m/s):0.86±0.04 (0.12)<br>**Post- measurements, week 13**<br>TANGOUPDRS-III:22.6±1.3 (3.9)<br>TUG:9.8±0.4 (1.2)<br>Gait velocity (m/s):0.88±0.04 (0.12)
Group 2 Dance	Hackney and Earhart, 2009 Short duration Tango	Uncontrolled Pilot Study Non Randomized	PD: n=12; 67.2 years; H&Y scale: 2.4)	**Duration:** 2 weeks, total 10 training sessions. 1.5-h., 5 days/week<br>PD= Intense programme of Argentine Tango lessons	**Means and SD**<br>Frequent social dance lessons completed within a short time period appear to be appropriate and effective for these individuals with mild-moderately severe Parkinson disease.<br>**Baseline**<br>UPDRS: 32.9 ±7.3<br>TUG(s):13.1 ± 5.8.<br>Gait Velocity(m/s): 1.0 ± 0.2<br>Step length(m): 0.6 ± 0.1<br>**Post-measurements**<br>UPDRS: 28.3 ± 7.1<br>TUG(s):11.1 ± 4.8<br>Gait Velocity (m/s):1.1 ± 0.2<br>Step length(m): 0.6 ± 0.1
Group 2 Dance	Heiberger et al., (2011) Narrative	Non-RCT	PD: n=11 (58–85 yr; 5 males)<br>H&Y scale II-IV<br>de novo: n=3 (66–71 yr; 2 males)<br>H&Y scale III–IV	**Duration:** 8 months, Dance class 1.15'h. once/week.<br>Mark Morris Dance class for Parkinson<br>Time Points:<br>- Short term-After class<br>-Long terms-after 8 months	The findings demonstrate that dance has beneficial effect on the functional mobility of individuals with PD. Further, dance improves the quality of life of the patients and their caregivers.

Group 2 Dance	Hackney and Earhard, 2009	RCT	WALTZ/FOXTROT: n = 17(males 11; 66.8 ± 2.4 years; H&Y scale: 2.0 ± 0.2)<br>TANGO: n= 14(males 11; 68.2 ± 1.4 years; H&Y scale:2.1 ± 0.1)<br>CTRL: n= 17 (males 12; 66.5 ± 2.8 years; H&Y scale: 2.2 ± 0.2)	**Duration:** 13 weeks, 2/week<br>WALTZ/FOXTROT: Progressive waltz and Foxtrot lessons.<br>TANGO: Progressive Argentine tango lessons.<br>CTRL: No intervention<br>**Time points:**<br>Pre- test<br>Post-test	**M and SE (SD)**<br>Tango may target deficits associated with PD more than Waltz/Foxtrot, but both dances may benefit balance and locomotion.<br>**Post- measurements, week 13**<br>WALTZ/FOXTROT<br>UPDRS III:24.3±3.4(14.01)<br>TUG(s): 10.8±1.2 (4.94)<br>Forward Velocity(m/s):<br>1.13 ±0.05(0.20)<br>Forward Stride length(m):<br>1.23 ±0.05 (0.20)<br>TANGO<br>UPDRS III:26.0 ±2.5 (9.35);<br>TUG(s):10.0 ±0.8 (2.99) Forward Velocity(m/s):1.19 ±0.05 (0.18) Forward Stride length(m):1.33 ±0.05(0.18)<br>CTRL<br>UPDRS III:32.4 ± 2.6 (10.7); TUG(s):14.4±2.6; Forward Velocity(m/s):1.09 ±0.10(0.41)<br>Forward Stride length(m):1.15 ±0.08(0.32).
Group 2 Dance	Volpe *et al.,*2013	Single blinded parallel group RCT	PD Irish Dance: n=12 (7 males 61.6 ± 4.5 years; H&Y scale: 2.2 ± 0.4)<br>PD Physiotherapy: n=12 (6 males 65.0 ± 5.3 years; H&Y scale: 2.2 ± 0.4)	**Duration:** 6 months; 90-min weekly. Watch a video dance 60-min once/week<br>PD Irish Dance=<br>70 minutes of Irish dance lessons and a 10 minutes cool down. Each person with PD was also given a video with recordings of the steps	**M and SD**<br>Both the Irish set dancing and physiotherapy exercise program were shown to be feasible and safe.<br>**Baseline**<br>PD Irish Dance<br>UPDRS-III: 24.58±3.87<br>PDQ-39: 30.60±12.06<br>**Post –measurements, 6 months**

				danced by the teacher. They were requested to watch the video at home once during<br>each week, for a period of 1 hour.<br>PD Physiotherapy=<br>designed to improve muscle strength, mobility, balance, and postural control according to the KNGF guidelines for physical therapy in Parkinson's disease. Each person with PD was given a video with recordings of the physiotherapy exercises. They were requested to watch the video at home once during each week, for a period of 1 hour.<br>**Time Points:**<br>Baseline (3 weeks prior to therapy)<br>Post-test (Discharge, within 3 weeks after the final week)	PD Irish Dance<br>UPDRS-III: 17.42±3.85<br>PDQ-39: 22.16±10.18
Group 2 Dance	Rios Romenets *et al.*, 2015	RCT	TANGO: n=18 (12 males; 63.2±9.9 years)<br><br>CTRL: n= 15 (12 males; 64.3±8.1 years)<br><br>H&Y scale: 1-3	**Duration:** 12 weeks; 60-min; 2/week<br>TANGO= Traditional Argentine Tango. The group continuous their physical activity but no new exercise programs or dancing classes<br>CTRL=self-directed exercise. The	**M and SD**<br>**TANGO**<br>Argentine tango can improve balance, and functional mobility, and may have modest benefits upon cognition and fatigue in Parkinson's disease.<br>**Baseline**<br>UPDRS-III:20.7±10.1<br>TUG(s):7.4±2.0

				group were provided<br>a pamphlet about exercise in PD (‘‘Exercises for people with Parkinson’s’’ Parkinson Society of Canada) and instructed to practice the exercises at home daily. If they were already<br>engaged in intensive regular exercise programs, they were allowed to continue their usual schedule of exercise<br>**Time points:**<br>Pre- test- week 0<br>Post - test-week 12	MoCa:27.0±2.4<br>PDQ-39:26.8±17.1<br>**Post-measurements**<br>UPDRS-III:19.1±10.2<br>TUG:6.1±1.5<br>MoCa:27.4±2.1<br>PDQ-39:26.4±18.9
Group 2 Dance	Hashimoto, *et al.,* 2015	RCT	DANCE: n=15 (3 males; 67.9 ± 7.0 years)<br>PD EXERCISE: n=17 (2 males; 62.7 ± 14.9 years)<br>CTRL: n=14 (7 males; 69.7 ± 4.0)<br>H&Y scale:2-4	**Duration:** 12 weeks 60-min session/week<br>DANCE: modern dance methods combinations of steps and movements from aerobic, jazz, tango dances, classical ballet, improvisation, and pantomime movements.<br>PD EXERCISE= physical therapy and PD exercises<br>presented by book or video (extending the range of joint motion in upper and lower body, maintaining balance, shifting the	**M and SD**<br>**Post-test-week 14**<br>Dance was effective in improving motor function, cognitive function, and mental symptoms in PD patients. General symptoms in PD also improved.<br>DANCE<br>TUG (time/s):9.7 ± 2.1 (P=0.006)<br>CTRL<br>TUG (time/s):10.2 ± 2.4 (P=0.006)

				center of gravity, walking on the spot, rising from and sitting down in a chair, and walking).<br>CTRL=participants continued with their normal life.<br>**Time points:**<br>Pre-test: a week prior intervention<br>Post- test: week 13	
Group 2 Dance	Westheimer *et al.*,2015	Non randomized Pilot study	Dance n=14 analyzed n= 12 (6 men, 66.2 ± 7.3yrs<br>H& Y I- IV	**Duration:** 8 weeks, total 16 sessions, 2/week<br>Dance classes= DfPD Mark Morris Basic ballet movements and steps, some modern, jazz, and tap moves were taught. Movement across the floor emphasized walking in a variety of ways including gliding, marching, waltz, and combinations of these.<br>**Time points:**<br>Pre-intervention<br>Post-intervention	**Means and SD**<br>There was a significant change from baseline to post-intervention in total UPDRS III, and no significant changes from<br>baseline to post-intervention in PDQ-39SI<br>**Baseline**<br>UPDRS-III: 28.8 ± 9.6<br>PDQ-39SI: 25.3 ±20.3<br>**Post-measurements, week 8**<br>UPDRS-III: 25.8±9.4<br>PDQ-39: 25.1±17.6
Group 2 Dance	Shanahan *et al.,* 2015 Narrative	Clinical Trial	IRISH DANCE GROUP: n= 9 (7 males, 66.66 ± 5.87 yrs)<br>H&Y scale:1-3	**Duration:** 8 weeks. 1.5 h set dancing class, Once/week<br>Class based on Irish dance.<br>**Time points:**<br>Pre- test<br>Post-test	**Medians and quartiles range(minimum-maximum)= Means and SD**<br>Quality of life improved with the dance programme and there was a trend toward improvement on the UPDRS-III. Community-based Irish set dancing is a feasible form of exercise that can positively influence quality of life.<br>**Baseline**<br>UPRRS-III: 11 ± 7.5 (6, 25) = 12.9 ±6.3

					PDQ-39: 23.30 ± 17.46 (4.59, 36.82)=22.17±10.8<br>**Post-measurements**<br>UPRRS-III :9 ± 8 (4, 18)=9.8±4.6<br>PDQ-39: 19.27 ± 15.93 (2.08, 31.41)=18.17±9.8<br>Note: Values are Medians and interquartile and are non-normal distributed. Not log transformed **Not in meta-analysis.**
Group 2 Dance	Shanahan *et al.,* 2017 Narrative	RCT pilot study	DANCE: n= 20 (13 males; 69±10 years)<br>CTRL: n=21(13 males; 69±8 years)<br><br>H&Y scale:1-3	**Duration:** 10 weeks, 1,5 h once/week and 20-min home dance 3/week<br>DANCE=1.5 hours Irish dance. Set dance combine steps and sequences of group movement patterns such as Connemara Set, Kilfenora set, and Corofin plain set. In addition participants were given a 20 minute home dance programme.<br>CTRL=Usual care and daily activities<br>**Time points:**<br>Pre- test<br>Post-test	**Medians and quartiles range (minimum-maximum)= Means and SD**<br>Post-intervention, the dance group had greater non-significant gains in quality of life than the usual care group. There were no meaningful changes in another outcome. Set dancing is feasible and enjoyable and may improve quality of life.<br>**Post measurements**<br>DANCE<br>UPDRS-III: 12.12 (6.36-22.12)=12.75±4.21<br>CTRL<br>UPDRS-III: 17.27(7.27-24.24) =16.8±4.49<br>Note: Values are not normal distributed and not log transformed. **Not in meta-analysis.**
Group 2 Dance	Kunkel, *et al.,* 2017	RCT	DANCE: n=36 (19 males; 49-85 years).<br><br>CTRL: n=15 (6 males; 59-80 years).<br><br>H&Y scale:1-3	**Duration**: 10 weeks, 60 min 2/week.<br>DANCE=3 ballroom dances (social foxtrot, waltz and tango) plus 3 Latin American, cha cha, rock-and-roll and rumba)<br>CTRL = Usual care, (medication,	**M and SD**<br>**Follow up-3 months**<br>Feasible program to conduct in a Dance Centre Participants<br>described dance as extremely enjoyable and the instructors were skilled in instilling confidence and motivation.

				attending medical clinics and routine visits from Parkinson's nurses)<br>**Time points**:<br>Baseline<br>Follow up:<br>a) 3 months<br>b) 6 months	DANCE<br>PDQ39:19.9 ±12.5<br>TUG: 14.4s ±5.4s<br>CTRL<br>PDQ39:18.3±10.8. TUG:12.5s±4.3s
Group 2<br>Dance	Lee *et al.,*<br>2018	RCT<br>PARTIAL<br>CROSS<br>OVER	TURO PD: n=25 (65.8±7.2 years)<br>CTRL: n=16 (67.7±6.4 years)<br>H&Y scale: 1-3	Duration: 8 weeks, 60-min., 2/week.<br>TURO PD=<br>combination of simple Qigong parts and<br>meditation exercises designed to be danced along with<br>music<br>CTRL=<br>Usual treatment<br>**Time Points:**<br>Pre- test<br>Post-test week 8	**M and SD.**<br>**Post- test-week 8**<br>Turo PD training<br>might improve the symptoms of PD patients.<br>TURO PD<br>UPDRS III: 13.8±6.9<br>PDQL total:144.6±20.9<br><br>CTRL<br>UPDRS III:128±4.2<br>PDQL total:132.6±22.3
Group 2<br>Dance	Mc Gill *et al.,*<br>2018<br>Narrative	Clinical<br>Trial	EX n=18 (69.83±4.55 yr, 9 males)<br>CTRL: n=13 (73.25±8.09 6 males)<br>H&Y scale: 1-3	Duration: 10-12 months 1/week, 1.25 and 1.5 h min.<br>EX=<br>ballet-based dance intervention<br>CTRL=<br>not involved in any dance classes<br>**Time Points:**<br>Pre- test<br>Post-test long term month 12	No significant effects of weekly ballet classes on gait variability or balance confidence. Findings differ from recent studies that suggest<br>dancing can improve balance and gait for this population.

Group 2<br>Dance	Rawson *et al.,*<br>2019	Clinical<br>Trial	TANGO: n= 39(66.73±9.52 years)<br>TREADMILL n= 31 (68.52±9.54 years)<br>ACTIVE CTRL -STRETCHING n=26<br>(66.18 ±7.30 years)	Duration: 12-weeks, 60-min<br>once/week<br>TANGO = Participants practiced<br>Argentine tango using an adapted<br>curriculum for persons with PD.<br>TREADMILL=<br>To approximate the intensity of<br>activity in the tango classes,<br>treadmill participants walked at<br>their preferred over-ground<br>walking speed. Treadmills were<br>arranged in groups of 4 (2 pairs<br>facing each other) to allow for<br>social interactions.<br>STRETCHING= gentle stretching<br>and whole-body flexibility exercises<br>designed for people with PD<br>**Time points:**<br>Baseline Post, week 12<br>Follow up, 12 weeks after post	**M and SE (SD)**<br>Forward velocity improved for the<br>treadmill group from baseline to posttest and<br>improvements persisted<br>at follow-up. There were no significant changes in the<br>tango group across time points.<br>**Baseline**<br>TANGO<br>Forward velocity (cm/sec): 104.53±3.9(24.48)<br>UPDRS-III: 36.92± 1.95 (12.17)<br>PDQ-39: 18.33 ± 1.89 (11.80)<br>**Post**<br>TANGO<br>Forward velocity (cm/sec): 103.36 ± 4.21(26.29)<br>UPDRS-III:35.23± 1.92 (11.90)<br>PDQ-39:18.80 ± 1.85 (11.55)
Group 2<br>Dance	Solla *et al.,*<br>2019	RCT<br>Pilot<br>study	BS: n=10(67.8± 5.9yrs)<br>CTRL: n=9 (67.1±6.3 yrs)<br>H&Y scale: 1-3	Duration: 12 weeks, 90-min.,<br>2/week.<br>BS=Ballus Sardu<br>CTRL=Usual care<br>**Time Points:**<br>Pre- test<br>Post-test week 12	**M and SD.**<br>**Post- measurements-week 12**<br>Ballus Sardu has been proved to be superior to usual<br>care alone in inducing changes in different motor and<br>non-motor symptoms associated with PD.<br>TURO PD<br>UPDRS III: 7.70±6.70<br>TUG (s): 5.08±0.78<br>Stride length (m): 1.33±0.10<br>Gait speed (m/s): 1.34± 0.09

					MOCA: 26.40±3.47<br>CTRL<br>UPDRS III: 15.55±6.25<br>TUG (s): 6.95±1.19<br>Stride length (m): 1.27±0.19<br>Gait speed (m/s): 1.20± 0.20<br>MOCA: 25.22±2.68
Group 3 Dance	Krishnamurthi et al., 2019	Clinical Trial	M&M:n=19 (8 males 66.7±7.2 yrs)<br>Analyzed n=15<br>H&Y scale 1-2	Duration: 10 weeks, 60min., 2/week<br><br>Dance based program<br><br>Time points:<br>Pre-test<br>Post-test (week 10)	M and SD<br><br>Regular practice of PD-specific Movement & Motion training can alleviate the targeted impairments and, thus, may lead to improved mobility and quality of life for people with PD.<br><br>Baseline<br>UPDRS—part III: 14.2±6.2<br>PDQ-39—total: 27.5±20.3<br>Velocity (m/s): 1.25±0.23<br>Stride length (m): 1.29±0.21<br><br>Post-measurements, week 10<br>UPDRS—part III: 13.1±8.6<br>PDQ-39—total: 23.7±19.7<br>Velocity (m/s): 1.36±0.24<br>Stride length (m): 1.34±0.21
Group 2 Dance	Poier et al., 2019	RCT	TANGO: n=14(9 males; 69±8years)<br>TAI-CHI: n=15(3 males; 69±11 years)	Duration: 10-weeks. 60min; once a week<br>TANGO=<br>Neurotango TM concept which combines preparatory	M and SD<br>No improvements were found in both groups for all outcomes.<br>Baseline<br>TANGO

				neuromotoric<br>exercises with adapted tango-specific movements.<br>TAI-CHI=<br>Tai Chi topics and forms e.g.: Tai Chi system, energy self-massage to stimulate the body, Tai Chi stepping/brush knees, ankle sway/weight shifting,<br>repulse monkey, and wave hands like clouds.<br><br>Time points:<br>Baseline<br>During intervention(middle)<br>Post-intervention (week 10)	PDQ-39: 25.06±9.82<br>Post measurements- week 10<br>TANGO<br>PDQ-39:27.29±10.48 (P=0.085)
Group 2 Dance	Kalyani et al., 2019	quasi experimental parallel group	DG: n=17(3 males; 65.7±11.88 years; H&Y scale: 1.6±0.7<br>CTRL: n=16 (10 males 66.50±7.70 years; H&Y scale: 1.5±0.81	Duration: 12 weeks. 60 min; 2/week.<br>DG=DfPD classes: aspects of ballet modern dance, choreographic repertory, Jazz, tap, Flamengo and Scottish dance.<br>CTRL=The usual treatment.<br>Time points: Pre-test<br>Post-test	**M and SD**<br>**Post-measurements , week 12**<br>Dance classes had a clear benefit on psychological symptoms, Quality of life and a limited cognitive benefit.<br>DG<br>PDQ-39:19.45± 12.45.<br>CTRL<br>PDQ-39: 16.82±10.14
Group 2 Dance	Kalyani et al., 2020	quasi-experimental controll	Dance group-DG n=17<br>CTRL n=16	Duration: 12 weeks, 60m 2/week<br>DG=DfPD classes<br>30-minute seated dance; 10-15 minutes each of standing dance	**Means and SD**<br>Post- measurements– week 12<br>DfPD®-based dance classes improved disease-related symptom severity, fine-manual dexterity, and functional

		ed study		with support, then standing dance with movement across the floor. Over time the intervention was progressively advanced to include more complex movements. caregiver.<br>CTRL= PD treatment as usual, which comprised individual routine clinical care.<br>Time points: Pre-test<br>Post-test	mobility.<br>DG<br>UPDRS –III: 23.24±14.35<br>TUG: 10.6±2.9 s<br>CTRL<br>UPDRS –III: 29.94±13.90<br>TUG: 11.4±3.3 s
Group 2 Dance	Dos Santos Delabary et al., 2020	Clinical Trial	DG: n= 12 (68.6 ± 6.7 years)<br>WG: n= 6 (64.2 ± 4.9 years)<br><br>H&Y scale: 1-3	Duration: 12 weeks total 24 sessions; 60 min 2/week<br>Two interventions; the first was preparatory and the second is the Dance program inspired by Samba and Forró rhythms<br>The walking program was performed outdoors on a 400m track to facilitate the calculation of the distance covered in each training session.<br>Time points:<br>Pre-test<br>Post-test, week 12	**Means and SE (SD)**<br>Functional mobility improved similarly in both groups. The results suggest that a 12-week program of Brazilian dance was sufficient to produce improvements in functional mobility and gait in individuals with PD.<br>DG<br>Baseline<br>TUG(s): (11.7±2.5) 11.70±2.42<br>Gait velocity (m/s): (1.02 ±0.05) 1.02 ±0.17<br>Post-measurements, week 12<br>TUG(s): (10.20±0.5) 10.20 ±1.73<br>Gait velocity (m/s): (1.10±0.07) 1.10 ±0.24
Group 2 Dance	Harrison et al., 2020	Non randomized Pilot	Dance n= 11 (69±8 years)<br>H&Y I-III	Duration: Total 6 weeks for a total of 12 classes;60 min 2/week<br>Contemporary dance intervention<br>Time points:	**Means and SD**<br>Significant increase in gait speed, cadence and stride length and a significant decrease in single support time variability. No significant changes in

		study		Pre-intervention<br>Post-intervention	other measures<br>of gait variability nor in motor symptoms, mood and anxiety, extent of life-space mobility, or quality of life.<br>Baseline<br>MDS-UPDRS-III:<br>29±13<br>PDQ-39: 23±13<br>Gait<br>Velocity (m/s): 1.33±0.27<br>Stride length<br>(m):1.25±0.21<br>Post-measurements, week 8<br>MDS-UPDRS-III:<br>38±14<br>PDQ-39: 23±15<br>Velocity (m/s): 1.42±0.29<br>Stride length (m): 1.31±0.20
Group 2<br>Dance	Tilmann et al., 2020	Clinical Trial	EX n= 20 (65.30±10.5 yrs)<br>CTRL n= 20<br>(67.6±10.9 yrs)<br>H& Y I-IV	Duration: 12 weeks; 60 min 2/week<br>EX= Samba dance classes<br><br>CRTL= no physical activity<br>Time points:<br>Pre-test<br>Post-test	**Means and SD**<br>Improvements in<br>the UPDRS global score, in daily activities, on motor examination in balance scores and in the mobility domain of the quality of life in the experimental group.<br>Post measurements<br>EX<br>UPDRS-III: 12.0±2.8<br>PDQ-39: 49.0±27.9<br>CRTL<br>UPDRS-III: 25.1±2.8<br>PDQ-39: 66.4±9.3
Group 3	Scandalis *et*	Clinical	PD: n= 14	**Duration:** 8-weeks; 2/week	**M and SD**

Strength	*al*., 2001	trial	(48-78 yrs)<br>CTRL: n= 6 Healthy adults<br>(58-67 yrs)<br>H&Y scale 2-3	Both groups received<br>rigorous resistance training<br>schedule.<br>The exercises were<br>geared toward the<br>lower body.<br>**Time points:**<br>Pre-test<br>Post -test	**PD**<br>Resistance training produce increases in strength<br>Resistance and functional improvements in gait.<br>**Baseline**<br>Lower limb strength (reps x weight)<br>Leg curl:151 ± 195<br>Leg extension: 207± 262<br>Leg press: 322±380<br>Gait<br>Stride length (m):<br>0.83 ±0.28<br>**Post-test- week 8**<br>Lower limb strength (reps x weight)<br>Leg curl:258 ± 329<br>Leg extension: 451± 525<br>Leg press: 620±716<br>Gait<br>Stride length (m)<br>0.95± 0.30
Group 3<br>Strength	Dibble *et al*.,<br>2006	Clinical<br>Trial	EX GROUP: n= 10<br>(64.3 ±9.6 yrs)<br>Analyzed n=1<br>CTRL<br>GROUP: n= 10 (67.0 ±10.2 yrs)<br>Analyzed n=9<br>40-85 years<br>H&Y scale 1-3	**Duration:** 12-weeks; 40-60min<br>3/week<br>Both groups participants engage<br>the same components of exercise<br>training<br>with the exception of differing in<br>the mode of lower<br>extremity resistance training only.<br>EX = standard care high-force	**M and SD**<br>High-force eccentric resistance training demonstrates<br>the ability to hypertrophy being<br>important in improving muscle force and<br>mobility in persons with PD**.**<br>**EC**<br>**Baseline**<br>Average torque (Nm)<br>Most affected side of the body:

				eccentric resistance training for traditional lower extremity resistance strength training.<br>CTRL =Standard care (rehabilitation program that includes calisthenics, stretching, walking, cycle ergometer, lifting weights.<br>**Time points:** Pre-training<br>Post -training	69.94 ± 23.98<br>**Post-test:**<br>90.74 ±31.03
Group 3 Strength	Dibble *et al.*, 2009	Non Randomized experimental study; Matched Pairs Design	EX: n=10.<br><br>CTRL: n=10.<br>62-82 years H&Y scale: 3-4	**Duration:** 12-weeks, 45-50 min., 3 days / weeks.<br>EX=Standard exercise+ additionally eccentric training for traditional lower extremity resistance training.<br>CTLR =Standard exercise<br>**Time points:** Baseline.<br>Post -test	**M and SD**<br>Significant time by group interaction effects for gait speed, timed up and go, and the composite PDQ-39 score. Muscle force, bradykinesia, and QOL were improved to a greater degree in those that performed high intensity eccentric resistance training compared to an active control group.<br>**EX**<br>**Baseline**<br>UPDRS- III:<br>Peak muscle force MO LE: 269.10±85.69<br>10min walk(m/s): 1.74 ±0.33. TUG (s): 7.13 ±2.19.<br>PDQ 39- Single Index: 51.89 ±16.38<br>**Post –week 12**<br>UPDRS-III: 9.00 ±4.60. Peak muscle force MO LE: 330.10±92.54<br>10min walk (m/s): 1.97 ±0.38.<br>TUG (s): 6.09 ±1.44.<br>PDQ 39- Single Index: 45.35 ±14.96,

Group 3 Strength	Hass et al., 2012	RCT	PRT: n=11<br>64 ±7 years<br>CTRL: n=11<br>67 ±8 years<br>H&Y scale: 1-3	**Duration:** 10 weeks-18 training sessions.<br><br>PRT=Progressive resistance training program<br>CTRL= continue their life style and not initiate any strength or balance training program.<br>**Time points:** Baseline.<br>Post -test	**M and SD**<br>**PRT**<br>The PRT group, participants significantly improved knee extension 1-RM strength by 76% and their knee flexion 1-RM strength by 57%<br>**Baseline**<br>**Strength 1-RM(kg)**<br>Knee extension: 43.0 ± 20.23<br>Knee Flexion: 26.4 ±10.94<br>**Post-test:**<br>Knee extension:75.9±28.19<br>Knee Flexion:41.5±14.59
Group 3 Strength	Carvalho *et al.,* 2015	RCT	AT: n=5 (64.8±11.9 years, H&Y scale: 2.6 ± 0.5<br>ST: n=8 (64.1 ±9.9years, H&Y scale: 2.1 ±0.6).<br>PST: n= 9 (62.1 ±11.7 years, H&Y scale: 2.3 ±0.5).	Duration: 12 weeks, 30 min., twice/week.<br>AT=walking (treadmill) 60% - 70% of VO2max.<br>ST=exercises for large muscle groups.<br>PST=30-40 min. calisthenics for the upper and lower limbs, stretching, and gait training.<br>**Time points:** Pre-test<br>Post-test-week 12	**M and SD.**<br>Improvements in disease symptoms and functional capacity.<br>**Baseline**<br>ST<br>UPDRS-III: 42.1 ±10.2<br>10-Minute Walk Test(s): 8.0±3.1.<br>**Post-test**<br>ST<br>UPDRS-III: 30.5±8.3;<br>10-Minute Walk Test(s):6.2±1.0.
Group 3 Strength	Silva-Batista *et al.,* 2016	Parallel RCT	RT: n=13 (64.1±9.1 years).<br>RTI: n=13 (64.2±10.6 years).<br>CTRL: n=13 (64.2±8.3 years).<br>H&Y scale: 2-3.	Duration: 12-weeks 50 min., twice / week.<br>RT=Resistance training; load/resistance exercises progressively increase.	**M and SD**<br>**Post-test**<br>Both training protocols improved muscle strength, but only RTI improved the mobility, motor signs, cognitive

				RTI=Resistance training; load/resistance progressively increase; plus, degree of instability.<br>CTRL=12 weeks, 60min once/week bingo games and lectures about PD.<br>**Time points**:<br>Pre-test<br>Post-test	impairment, and quality of life,<br>RT<br>PDQ-39:<br>40.3±9.16.<br>TUG:8.74±1.85.<br>UPDRS-III:43±12.86.<br>LP:113.22±25.4*<br>CTRL<br>PDQ-39:<br>42.59±14.2.<br>TUG:10.13±2.38<br>UPDRS-III:45.36±7.84<br>LP:85.18±25.93
Group 3 Strength	Silva-Batista *et al.,* 2017<br><br>Narrative	Parallel group RCT	RT: n=11 (8 men 64.4±9.1 yrs).<br>CTRL: n=11 (8 men 64.6±9.7 yrs).<br>HC: n=31 (13 men 64.6±8.4 yrs).<br>H&Y scale: 2-3.	Duration: 12-weeks 50 min., 2/ week<br>RT=Resistance training<br>CTRL=No training, 12 weeks Bingo games 60min 1/week<br>HC= no training<br>**Time points**:<br>Pre-test<br>Post-test	**M and SD**<br>RT improved sleep quality and knee-extensor peak torque of subjects with PD.<br>**Post-test**<br>PSQI-sleep disorders<br>RT: 4.2±0.8<br>CTRL:8.3±1.3<br>Knee extension Peak torque (N*m)<br>RT: 108±28<br>CTRL:67±29<br>The only study with sleep disorders
Group 3 Strength	Morris *et al.*,2017	RCT	EX: n=67<br>CONT: n=66<br>70.6 yr<br>H&Y scale: 1-4.	Duration: 6-week, 60min., twice/week.<br>EX=Home program with Progressive	**Means**<br>**Post-test, Week 6**<br>There were no differences between groups in UPDRS-II and health-related quality of life.

				resistance strength training, & movement strategy training and falls education.<br>CTRL=non-specific life skills training.<br>**Time points**: Baseline<br>Post-test, week 6<br>Follow up, 12 months	EX<br>UPDRS-III:28±14<br>PDQ-39:21±14<br>CTRL<br>UPDRS-III:30±13<br>PDQ-39:20±14
Group 3 Strength	Santos *et al.,* 2017.	RCT	EX: n=13 (5 men; 73.38± 8.81 years; H&Y scale: 1.92±0.42).<br>CTRL: n=15 (73.80±7.05; H&Y scale: 1.86±0.35).	Duration: 8 weeks, 60-70min. twice/week and non-consecutive dates.<br>EX and CTRL followed the usual physical activity routine.<br>EX=Progressive Resistance Exercise<br>**Time points:**<br>Pre-test<br>Post-test-week 8<br>Re-test- week 12	**M and SD**<br>PRE training in the rehabilitation of individuals with AR-subtype PD, as it can improve static posturography, gait, and quality of life.<br>**EX**<br>**Baseline**<br>TMWT(m/s) preferred rhythm:<br>0.87±0.15<br>PDQ-39:1.16±7.39<br>Speed (mm/s): 3.95±0.88<br>**Post-test –week 8**<br>TMWT(m/s) preferred rhythm:<br>0.85 ±0.15<br>PDQ-39:4.58±4.37<br>Speed (mm/s) :3.66±1.01
Group 3 Strength	Demonceau et al., 2017	Pseudo-randomized controlled trial.	Aerobic group n=16 (65±8 yr)<br>Strength group: n=15 (67±10 yr)<br>CTRL group: N=15 (63.3±6 yr)<br>H&Y scale 1-3	**Duration:** 12 weeks 60-90 min., twice to thrice / week<br>AE Group: aerobic training (cycling)<br>ST GROUP: exercises performed on machines or using weights.	**M and SD**<br>**Pre and post measurements**<br>Physical fitness in patients with PD rapidly improved in compliance with training specificities, but better fitness hardly translated into better mobility and health-

				CTRL GROUP: Standard care (includes exercise)<br>**Time points:**<br>Pre-intervention<br>Post-intervention- week 12.	related quality of life.<br>**ST**<br>**Baseline**<br>GAIT<br>Speed (m.s-1):1.16± 0.2<br>Stride length (m): 1.24 ± 0.19<br>TUG(s): 1.9±0.4<br>PDQ-39:24±12<br>Knee extension<br>Torque is set 60 $.s^{-1}$<br>Peak Torque- most effected ($Nm.kg^{-1}$): 1.488± 0.528<br>Knee flexion<br>Peak Torque- most effected ($Nm.kg^{-1}$): 0.636 ± 0.25<br>**Post**<br>Speed (m.s-1):1.22 ± 0.1<br>Stride length (m): 1.27 ± 0.14<br>TUG(s): 1.8 ± 0.3<br>PDQ-39:19±7<br>Knee extension<br>Peak Torque- most effected ($Nm.kg^{-1}$): 1.736 ± 0.453<br>Knee flexion<br>Peak Torque- most effected ($Nm.kg^{-1}$): 0.789 ± 0.277
Group 3<br>Strength	Ferreira et al., 2018	RCT	RTG: n=18<br>(64.1±7.0 years)<br>CTRL: n=17<br>(69.6 ±8.9 years)<br>H&Y scale: 1-3	**Duration:** 6 months 30-40 min.<br>Twice /week<br>RTG= Resistance Training Program<br>8-12 submaximal repetitions<br>CTRL=standard pharmacological treatment	**M and SD**<br>**Post-test, month 6**<br>Resistance training was effective in reducing anxiety symptoms and improved quality of life in patients with PD.<br>RTG

				**Time points:**<br>Pre-test<br>Post- test (week 12)	PDQ-39: 31.72± 19.75<br>CTRL<br>PDQ-39:47.24 21.34
Group 3 Strength	De Lima et al., 2019	RCT	RTG: n= 17 (66.2±5.5 years)<br><br>CTRL: n=16 (67.2±5.2 years)<br>H&Y scale: 1-3	**Duration:** 20 weeks with 30-40 min. 2 /week<br>RT= Resistance training protocol using<br>a combination of free weights and machines.<br>CTRL= No activity just the standard pharmacological treatment for PD.<br>**Time points:**<br>Pre-test<br>Post- test	**M and SD**<br>**Post-test, week 20**<br>Secondary outcomes<br>Resistance training reduces depressive symptoms and improves the<br>quality of life and functionality of elderly with PD.<br>RTG<br>TUG:26.4±5.5<br>PDQ-39:30.2±16.8<br>Gait Speed (m/s): 1.25 ± 0.34<br>CTRL<br>TUG=34.2±6.9<br>PDQ-39=40.6±15.6<br>Gait Speed (m/s): 0.91 ± 0.19
Group 3 Strength	Cherup *et al.*, 2019	RCT	ST: n= 21 (analyzed 18)<br>PT: n=21 (analyzed 17)<br>(40-70<br>H&Y scale: 1-3)	**Duration:** 12-weeks 60min. Twice /week<br>ST= high-load, low velocity strength training<br>PT=low-load, high-velocity power training<br>**Time points:**<br>Pre-test<br>Post- test (week 12)	**M and SD**<br>Both ST and PT appear to be effective at reducing the neuromuscular deficits associated with PD but not functional performance.<br>**ST**<br>**Baseline**<br>Leg Press 1RM(Kg):<br>122.2±11<br>TUG:7.96 ±3.58

					**Post**<br>Leg Press 1RM(Kg):<br>176±13<br>TUG:8.75 ±5.49
Group 3<br>Strength	Leal et al.,<br>2019	RCT	RT: n= 27 (14 men 64.9 ±2.32 years)<br>CTRL: n=27 (13 men 64.2±2.05 years)<br>H&Y scale: 1-3	**Duration:** 24 weeks with 30-40 min., 2 /week<br>RT= Resistance training protocol<br><br>CTRL= No activity just the standard pharmacological treatment for PD.<br>**Time points:**<br>Pre-test<br>Post- test	**M and SD**<br>**Post-test, week 24**<br>Low-volume resistance training improves the physical capacity.<br>TUG<br>RT:14.6±3.7<br>CTRL:18.7±4.3
Group 3<br>Strength	Vieira de Moraes Filho et al., 2020	RCT	TG: n= 31 (20 men 64.7 ±1.8 yrs)<br>Post analysis n=25<br><br>CTRL: n=31 (10 men 64.4±3.7 yrs)<br>Post analysis n=15<br>H&Y scale: 1-3	**Duration:** 9 weeks with 50-60 min., 2 /week<br>TG=Progressive resistance training with weight machines<br>CTRL= disease lectures<br>**Time points:**<br>Pre-test<br>Post- test	**M and SD**<br>**Post-test, week 9**<br>PRT reduces bradykinesia and improves functional performance in patients with mild to moderate PD.<br>TG<br>TUG(s): 7.4 ±2<br>TWM (m/s):2.0±1.5<br>Peak Torque (N·m): 147.1±38.5<br>Peak Torque (N·m/kg):<br>2.0±0.5<br>CTRL<br>TUG(s): 10.0±2.7<br>TWM (m/s): 1.7± 1.16<br>Peak Torque (N·m): 135.8±43.3<br>Peak Torque (N·m/kg): 1.8±0.77
Group 3<br>Strength	Strand et al.,<br>2017	Prospec tive	SPH: n= 17 (9 men<br>70.19±9.06 yrs) Analyzed n=13	Duration: 12 weeks with 60min. 3/week	**M and SD**<br>Pre and Post-test.

		parallel group RCT	SP+Func: n=18 (11 men 68.63±10.54 yrs)<br>Analyzed n=15<br><br>H&Y scale: 1-3	SPH=Strength and hypertrophy protocol<br>SP+Func=Strength and Functional protocol<br>Time points:<br>Pre-test<br>Post- test (week 12)	Both exercise strategies can be equally effective at improving functional capacity, balance, and muscular strength in individuals with PD.<br>Pre<br>SPH<br>TUG(s): 8.64 ±2.9<br>UPDRS-III: 28.85±6 12.91<br>PDQ-39: 21.69±14.20<br>LP1RM (kg): 181.6 ±81.7<br>Post<br>SPH<br>TUG(s): 7.76 ± 3.28<br>UPDRS-III: 24.46 ± 10.81<br>PDQ-39: 18.80± 11.05<br>LP1RM (kg): 206.4± 83.2

H&Y; Hoehn and Yahr Parkinson Disease Scale. CT: Control Trial. EX: experimental Group. SPT: Self Pace Training Group. NT: No Training Group. ON: on medication Group: OFF: off medication group. CTRL: Control Group. PD: Parkinson Disease Group. ICE: immediate carry over effects. DCE: Delay carry over effects. PrCrTs: pragmatic single blind randomized cross over trial. RAS: Rhythmic Auditory Stimulation. Non-RAS: without Rhythmic Auditory Stimulation. FT: Finger Taping training group. AS: Arm Swing group. MCT: Music Contingent Training group. ERAS:=Ecological rhythmic auditory stimulation. ARAS: Artificial auditory stimulation. BG = Binary Group. QG = Quaternary Group. EX: experimental group. MA LE: More Affected Lower Extremity. LA LE: Less Affected Lower Extremity. PRT: Progressive resistance training. RCIS: Randomize Controlled Interventional Study. AT: Aerobic Training Group. ST: Strength Training Group. P: Physiotherapy Group. PWT: Power-based resistance Training group. RT: Resistance Training Group. RTI: Resistance Training Instability Group. LP: Leg press. HC = age-matched healthy controls. PSQI= Pittsburgh Sleep Quality Index. AT: Aerobic Training Group. ST: Strength Training Group. PWT: Power-based resistance Training group. PT: Power Group. TG= Training group. PT=Peak Torque. SPH=strength, power, and hypertrophy training group; SP+Func= strength, power, and functional training group.

Table 2: Randomized control trials (RCTs) Risk of bias assessment outcomes
Key: **+** : low, **?** : some concerns, **-** : high, **N**: not applicable.

	First Author	Year Publication	Bias Randomization process	Bias Intervention assignment	Bias Intervention Adherence	Bias Missing Data	Bias Outcome	Bias Reported Results	Results
Rhythm	Thaut	1996	---	---	---	++	++	---	High
	De Bruin rhtyhm	2010	?	++	---	++	++	++	Some concerns
	Kadivar	2011	?	++	++	++	?	++	Some concerns
	Elston	2011	++	++	---	++	++	++	Some concerns
	De Icco	2015	?	++	---	++	?	++	High
	Thaut	2018	++	?	++	++	++	++	Low
	Murgia	2018	++	++	++	++	++	++	Low
	Braun Janzen	2019	?	++	++	++	++	++	Low
Dance	Hackney	2007	?	++	?	++	++	++	Some concerns
	Hackney& Earhart -E	2009	++	---	---	++	++	++	Some concerns
	Volpe	2013	++	?	?	++	++	++	Some concerns
	Rios Romenets	2015	++	++	?	++	?	?	Some concerns
	Hashimoto	2015	++	++	++	++	?	++	Low
	Kunkel	2017	?	?	?	++	++	++	Some concerns
	Poier	2017	++	?	---	?	?	++	High
	Sanahan	2017	++	++	++	?	?	++	Some concerns
	Lee	2018	?	++	++	++	++	++	Some concerns
	Solla	2019	++	++	++	++	++	++	Low
Strength	Hass	2012	?	++	++	++	++	++	Some concerns
	Carvalho	2015	?	++	++	++	++	++	Some concerns
	Silva-Batista	2016	?	++	++	++	++	++	Some concerns
	Silva - Batista	2017	?	++	++	?	++	++	Some concerns
	Morris	2017	++	?	?	++	++	++	Some concerns
	Santos	2017	++	++	++	++	++	++	Low
	Ferreira	2018	++	++	++	++	++	++	Low
	Cherup	2019	++	++	++	++	++	---	High
	Leal	2019	++	++	++	++	++	++	Low
	De Lima	2019	++	++	++	++	++	++	Low
	Vieira de Moraes Filho	2020	?	++	?	++	++	++	Some concerns
	Strand	2021	++	++	?	++	++		Low

Figure 1: RCTs Risk of bias assessment outcomes

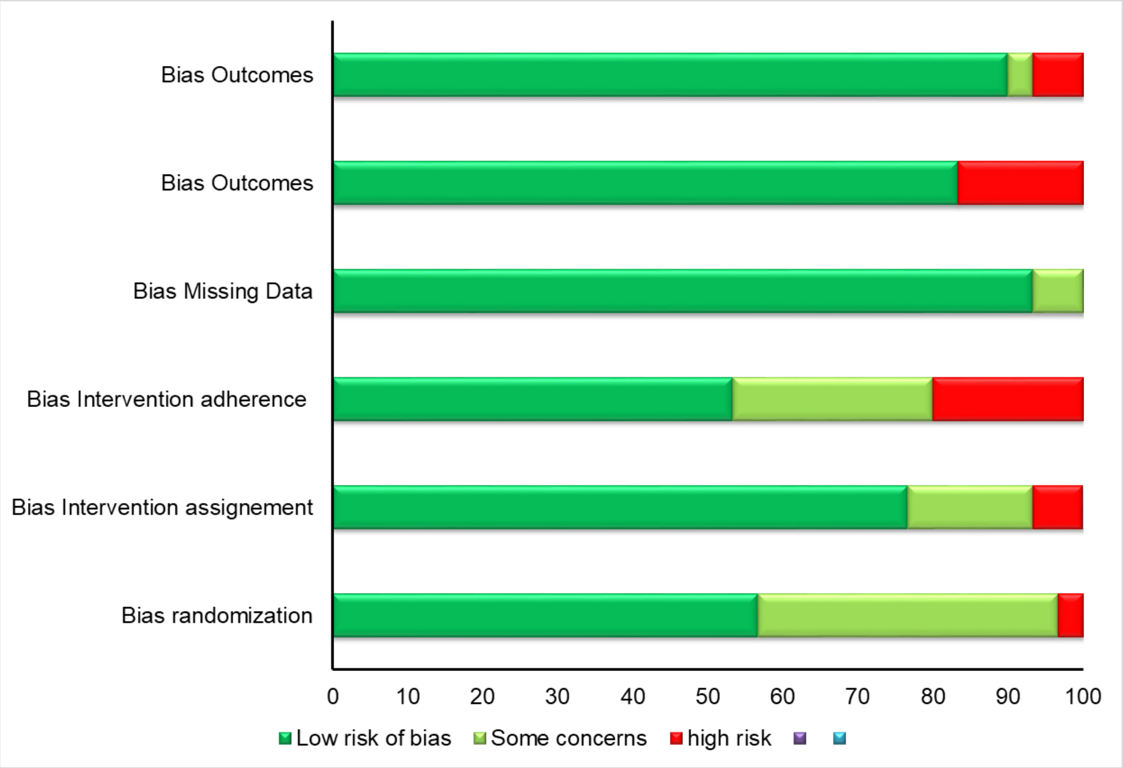

Table 3: Clinical Trials (CTs) Risk of bias assessment outcomes Key: ++: low, +: moderate, **?** : some concerns, **-** :serious, -- :critical, --- : high **NI** : no information.

	First Author	Year of publication	Confsounding	Bias Selection	Bias Classification	Bias Deviation of intervention	Bias Missing Data	Bias Outcomes	Bias Reported Results	Overall results
RHYTHM	McIntosh	1997	++	+	+	++	++	+	+	Moderate
	Ford	2010	++	+	++	++	++	+	++	Moderate
	Hausdorff	2017	++	+	+	++	++	+	++	Moderate
DANCE	Hackney and Earhart Short	2009	++	++	+	++	+	+	+	Low
	Heiberger	2011	++	++	++	++	++	+	+	Moderate
	Sanahan	2015	++	++	++	++	++	++	++	Low
	Westheimer	2015	++	++	++	++	++	++	++	Low
	McGill	2018	+	+	++	++	++	+	++	Moderate
	Rawson	2019	++	+	++	++	++	++	++	Moderate
	Kalyani	2019	++	+	+	++	++	+	++	Moderate
	Kalyani	2020	++	-	+	++	++	+	++	Serious
	Dos Santos Delabary	2020	++	+	++	++	++	+	++	Moderate
	Harrison	2020	++	++	++	++	++	++	++	Low
	Krishnamurthi	2020	++	++	++	++	+	++	++	Low
	Tillmann	2020	++	++	++	++	++	+	++	Low
STRENGTH	Scandalis	2001	++	+	+	++	++	?	++	Moderate
	Dibble	2006	++	+	++	++	++	+	++	Moderate
	Dibble	2009	++	++	+	++	++	+	++	Moderate
	Demonceau	2017	+	++	++	++	+	+	++	Moderate

Figure 2: CTs Risk of bias assessment outcomes

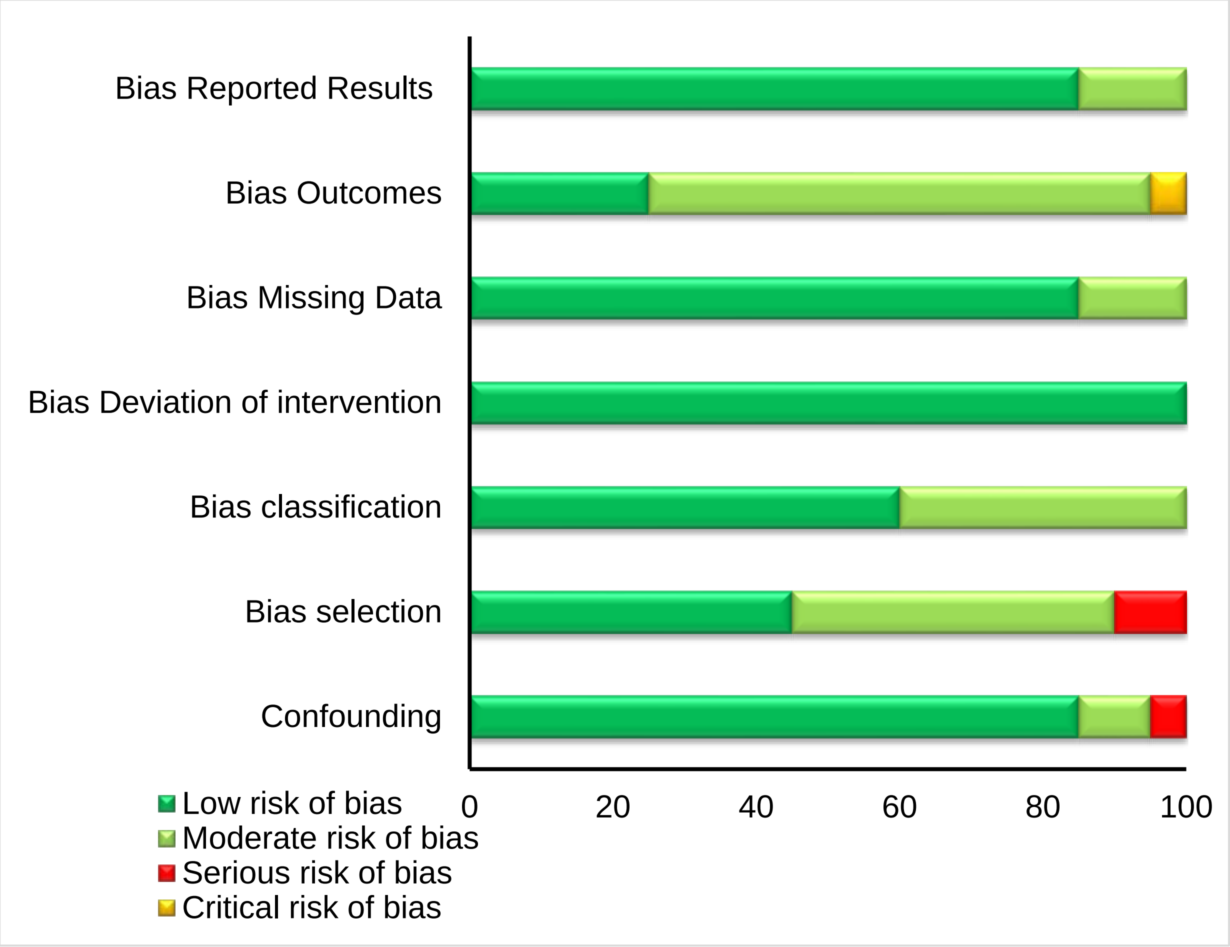

Figure 3: Forest plots of significant effects of rhythmic cueing on PD

3A: Forest plot of significant effects of rhythmic cueing on gait velocity

	Exercise RAS			Control				Std. Mean Difference
**Study or Subgroup**	**Mean**	**SD**	**Total**	**Mean**	**SD**	**Total**	**Weight**	**IV, Random, 95% CI**
Murgia et al., 2018 Art RAS	1.11	0.24	16	1.05	1.05	16	12.0%	0.08 [-0.62, 0.77]
Murgia et al., 2018 EcoRAS	1.21	0.25	16	1.07	1.07	16	12.0%	0.18 [-0.52, 0.87]
Brawn Janzen et al., 2018 Arm Swing	66.79	14.93	14	64.2	0.51	12	10.6%	0.23 [-0.55, 1.00]
Hausdorff et al., 2007	1.06	0.24	29	1	0.21	29	15.6%	0.26 [-0.25, 0.78]
De Bruin et al., 2010	1.31	0.22	11	1.25	0.17	11	9.6%	0.29 [-0.55, 1.13]
De Icco et al.,2015	0.77	0.3	11	0.63	0.22	11	9.5%	0.51 [-0.34, 1.36]
Thaut et al., 2018	65	13.2	25	53	16.8	22	13.9%	0.79 [0.19, 1.38]
Thaut et al, 1996	58.3	12.6	15	38.7	16	11	9.2%	1.34 [0.47, 2.22]
Brawn Janzen et al., 2018 Finger Tap	76.03	9.2	11	64.2	0.51	12	7.7%	1.79 [0.80, 2.79]
**Total (95% CI)**			**148**			**140**	**100.0%**	**0.54 [0.21, 0.88]**

Heterogeneity: Tau² = 0.12; Chi² = 14.73, df = 8 (P = 0.06); I² = 46%

Test for overall effect: Z = 3.20 (P = 0.001)

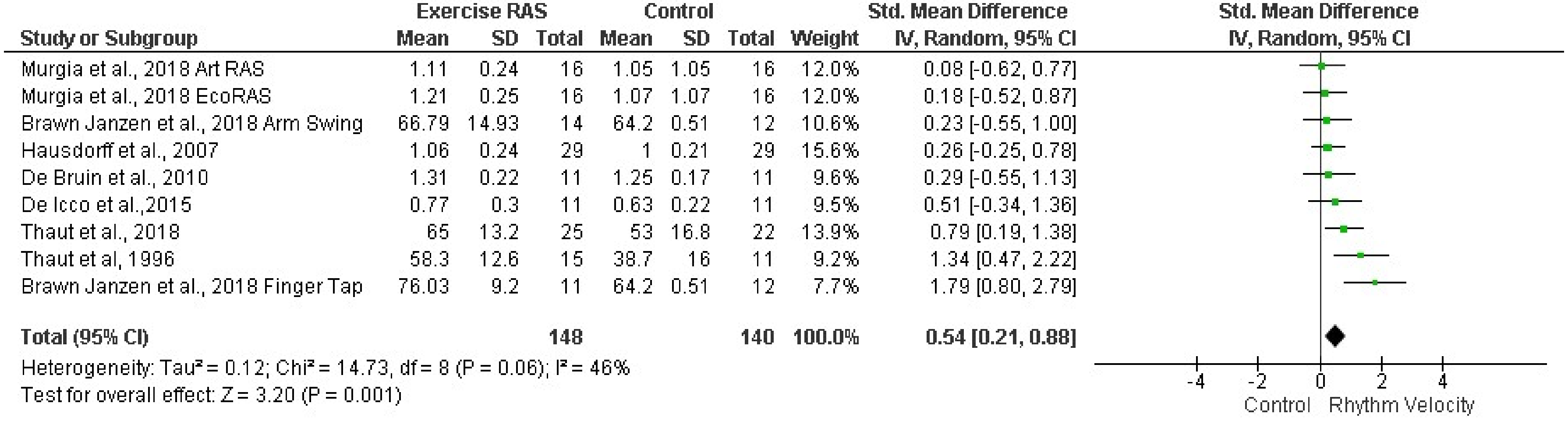

3B: Forest plot of significant effects of rhythmic cueing on stride length

	Exercise RAS			Control				Mean Difference
Study or Subgroup	Mean	SD	Total	Mean	SD	Total	Weight	IV, Random, 95% CI
Thaut et al, 1996	1.1	1.7	15	1.12	0.21	11	0.4%	-0.02 [-0.89, 0.85]
Murgia et al., 2018 Art RAS	1.16	0.18	16	1.13	0.2	16	12.3%	0.03 [-0.10, 0.16]
Murgia et al., 2018 EcoRAS	1.2	0.19	16	1.16	0.2	16	11.9%	0.04 [-0.10, 0.18]
Hausdorff et al., 2007	1.12	0.2	29	1.06	0.21	29	15.8%	0.06 [-0.05, 0.17]
De Bruin et al., 2010	1.37	0.18	11	1.3	0.14	11	11.9%	0.07 [-0.06, 0.20]
Brawn Janzen et al., 2018 Arm Swing	1.25	0.179	14	1.17	0.159	12	12.5%	0.08 [-0.05, 0.21]
Brawn Janzen et al., 2018 Finger Tap	1.29	0.135	11	1.17	0.159	12	13.7%	0.12 [-0.00, 0.24]
Thaut et al., 2018	1.19	0.14	25	1.01	0.14	30	21.5%	0.18 [0.11, 0.25]
De Icco et al.,2015	106.7	10.7	8	83.5	25.7	8	0.0%	23.20 [3.91, 42.49]
**Total (95% CI)**			**145**			**145**	**100.0%**	**0.09 [0.03, 0.15]**

Heterogeneity: $Tau^2 = 0.00$; $Chi^2 = 12.79$, df = 8 ($P = 0.12$); $I^2 = 37\%$
Test for overall effect: $Z = 3.08$ ($P = 0.002$)

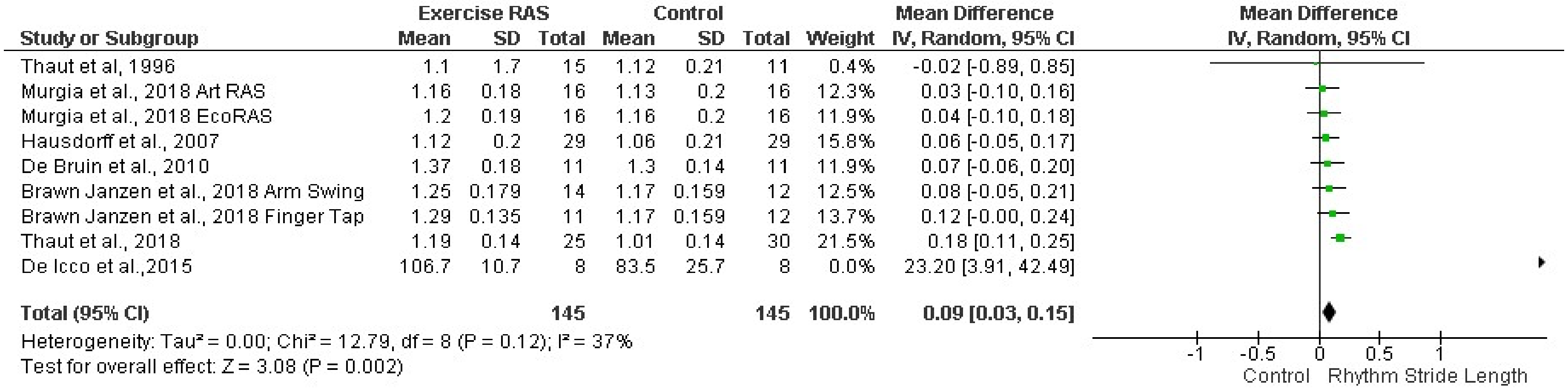

3C Forest plot of significant effects of rhythmic cueing on motor symptoms-UPDRS-III

Study or Subgroup	Exercise RAS Mean	Exercise RAS SD	Exercise RAS Total	Control Mean	Control SD	Control Total	Weight	Mean Difference IV, Random, 95% CI
De Icco et al.,2015	24.1	9.3	8	32.1	9.8	8	13.6%	-8.00 [-17.36, 1.36]
Murgia et al., 2018 EcoRAS	11.64	7	16	16.93	9.14	16	35.1%	-5.29 [-10.93, 0.35]
Murgia et al., 2018 Art RAS	15.36	9.2	16	20.21	9.59	16	27.0%	-4.85 [-11.36, 1.66]
De Bruin et al., 2010	19.9	9.05	11	18.6	7.38	11	24.3%	1.30 [-5.60, 8.20]
**Total (95% CI)**			**51**			**51**	**100.0%**	**-3.94 [-7.47, -0.41]**

Heterogeneity: Tau² = 0.96; Chi² = 3.23, df = 3 (P = 0.36); I² = 7%
Test for overall effect: Z = 2.19 (P = 0.03)

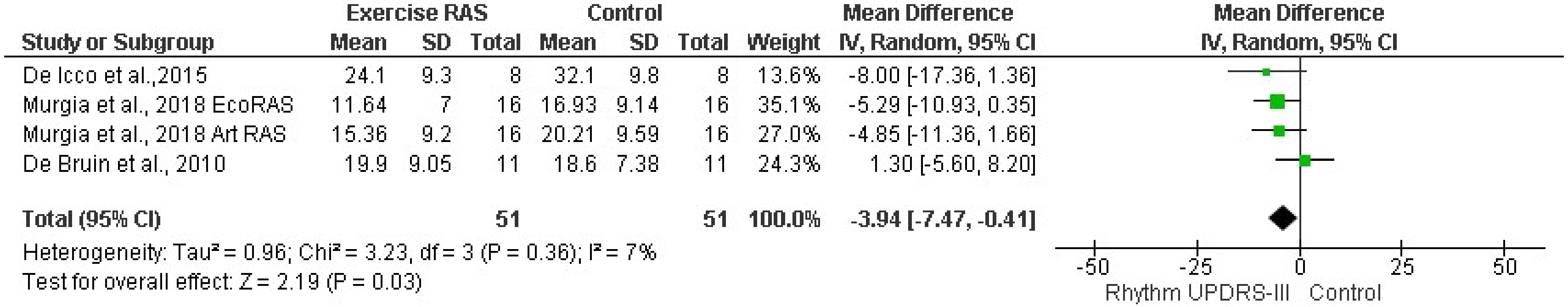

Figure 4: Forest plots of significant effects of dance on Parkinson disease

4A: Forest plot of significant effects of dance on stride length

Study or Subgroup	Experimental Mean	Experimental SD	Experimental Total	Control Mean	Control SD	Control Total	Weight	Mean Difference IV, Random, 95% CI
Hackney and Earhart, 2010	1.09	0.2	15	1.08	0.3	19	19.2%	0.01 [-0.16, 0.18]
Solla et al., 2019	1.33	0.1	10	1.27	0.19	9	28.3%	0.06 [-0.08, 0.20]
Harrison et al., 2020	1.31	0.2	11	1.25	0.21	11	18.6%	0.06 [-0.11, 0.23]
Hackney and Earhart, 2009 a waltz	1.23	0.2	17	1.15	0.32	17	16.9%	0.08 [-0.10, 0.26]
Hackney and Earhart, 2009 a Tango	1.33	0.18	14	1.15	0.32	17	17.0%	0.18 [0.00, 0.36]
**Total (95% CI)**			**67**			**73**	**100.0%**	**0.07 [0.00, 0.15]**

Heterogeneity: Tau² = 0.00; Chi² = 1.97, df = 4 (P = 0.74); I² = 0%
Test for overall effect: Z = 1.97 (P = 0.05)

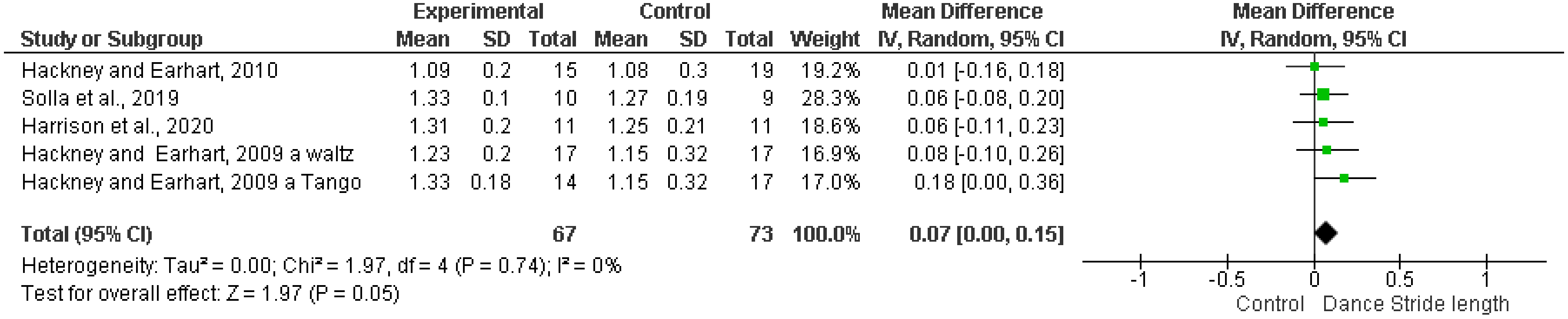

4B: Forest plot of significant effects of dance on functional mobility-TUG

Study or Subgroup	Experimental Mean	Experimental SD	Experimental Total	Control Mean	Control SD	Control Total	Weight	Mean Difference IV, Random, 95% CI
Hackney and Earhart, 2009 a Tango	10	2.99	14	14.4	10.72	17	0.9%	-4.40 [-9.73, 0.93]
Hackney and Earhart, 2009 a waltz	10.8	4.94	17	14.4	10.72	17	0.8%	-3.60 [-9.21, 2.01]
Hackney and Earhart, 2009 c Short Duration	11.1	4.8	12	13.1	5.8	12	1.4%	-2.00 [-6.26, 2.26]
Solla et al., 2019	5.08	0.78	10	6.95	1.19	9	31.2%	-1.87 [-2.79, -0.95]
Dos Santos Delabary et al., 2020	10.2	1.73	12	11.7	2.42	12	9.2%	-1.50 [-3.18, 0.18]
Rios Romenets et al., 2015	6.1	1.5	11	7.4	2	18	15.9%	-1.30 [-2.58, -0.02]
Hackney et al., 2007	9.8	1.2	9	10.7	1.2	9	21.3%	-0.90 [-2.01, 0.21]
Kalyani et al., 2020	10.6	2.9	17	11.4	3.3	16	5.8%	-0.80 [-2.92, 1.32]
Hashimoto, et al., 2015	9.7	2.1	15	10.2	2.4	14	9.6%	-0.50 [-2.15, 1.15]
Hackney and Earhart, 2010	13.2	9	15	13.6	9	19	0.7%	-0.40 [-6.49, 5.69]
Kunkel, et al., 2017	14.4	5.4	30	12.5	4.3	15	3.1%	1.90 [-1.01, 4.81]
**Total (95% CI)**			**162**			**158**	**100.0%**	**-1.26 [-1.77, -0.75]**

Heterogeneity: $Tau^2 = 0.00$; $Chi^2 = 9.91$, $df = 10$ ($P = 0.45$); $I^2 = 0\%$
Test for overall effect: $Z = 4.82$ ($P < 0.00001$)

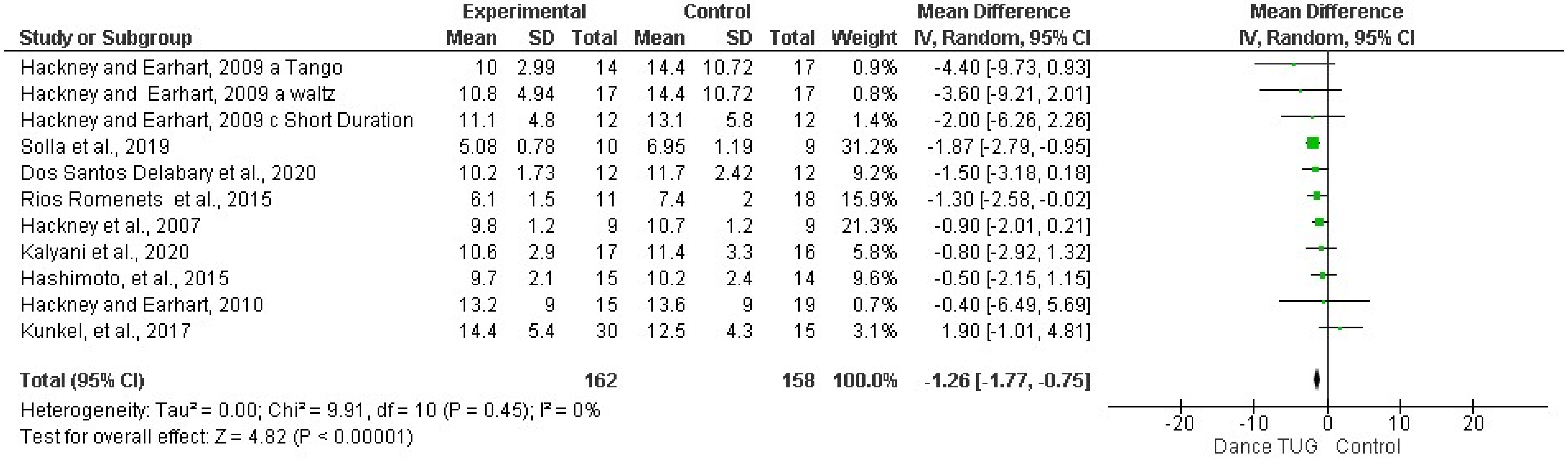

4Ba: Funnel Plot of significant effects of dance on functional mobility-TUG

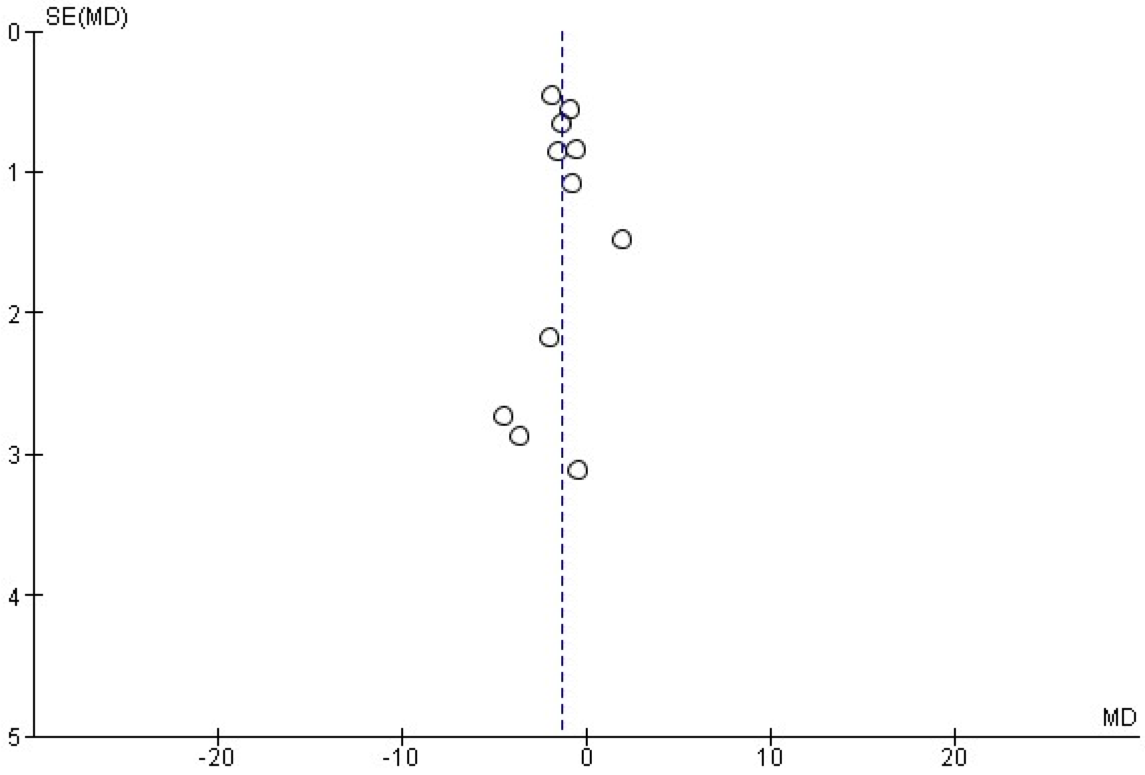

4C: Forest plot of significant effects of dance on motor symptoms-UPDRS-III

Study or Subgroup	Experimental Mean	Experimental SD	Experimental Total	Control Mean	Control SD	Control Total	Weight	Mean Difference IV, Random, 95% CI
Tilmann et al., 2020	12	2.8	20	25.1	2.8	20	10.9%	-13.10 [-14.84, -11.36]
Hackney and Earhart, 2009 a waltz	24.3	14.01	17	32.4	10.72	17	6.1%	-8.10 [-16.49, 0.29]
Hackney et al., 2007	22.6	3.9	9	30.6	3.9	9	9.8%	-8.00 [-11.60, -4.40]
Solla et al., 2019	7.7	6.7	10	15.55	6.25	9	8.0%	-7.85 [-13.67, -2.03]
Volpe et al.,2013	17.42	3.85	12	24.58	3.87	12	10.1%	-7.16 [-10.25, -4.07]
Kalyani et al., 2020	23.24	14.35	17	29.94	13.9	16	5.3%	-6.70 [-16.34, 2.94]
Hackney and Earhart, 2009 a Tango	26	9.35	14	32.4	10.72	17	7.1%	-6.40 [-13.47, 0.67]
Hackney and Earhart, 2009 c Short Duration	28.3	7.1	12	32.9	7.3	12	8.1%	-4.60 [-10.36, 1.16]
Westheimer et al.,2015	25.8	9.4	12	28.8	9.6	12	6.7%	-3.00 [-10.60, 4.60]
Rawson et al., 2019	35.23	11.9	39	36.92	12.17	39	8.4%	-1.69 [-7.03, 3.65]
Rios Romenets et al., 2015	19.1	10.2	11	20.7	10.1	18	6.6%	-1.60 [-9.22, 6.02]
Krishnamurthi et al., 2019	13.1	8.6	15	14.2	6.2	15	8.4%	-1.10 [-6.47, 4.27]
Harrison et al., 2020	38	14	11	29	13	11	4.5%	9.00 [-2.29, 20.29]
**Total (95% CI)**			**199**			**207**	**100.0%**	**-5.38 [-8.44, -2.32]**

Heterogeneity: Tau² = 21.60; Chi² = 57.55, df = 12 (P < 0.00001); I² = 79%
Test for overall effect: Z = 3.44 (P = 0.0006)

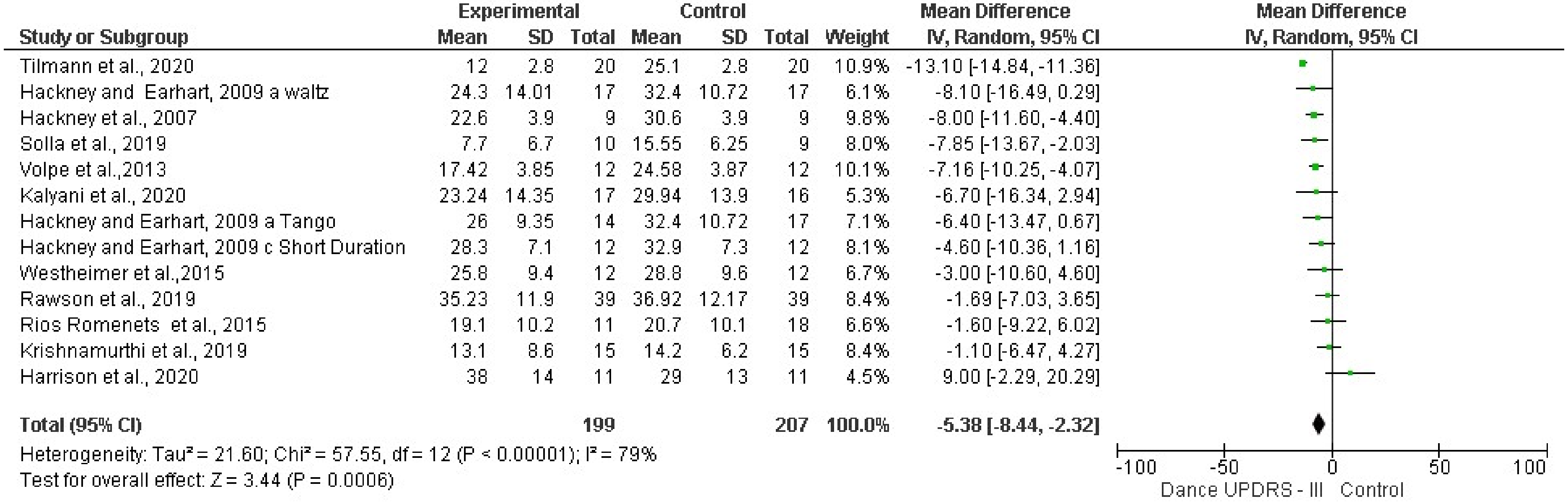

4Ca: Funnel plot of significant effects of dance on motor symptoms-UPDRS-III

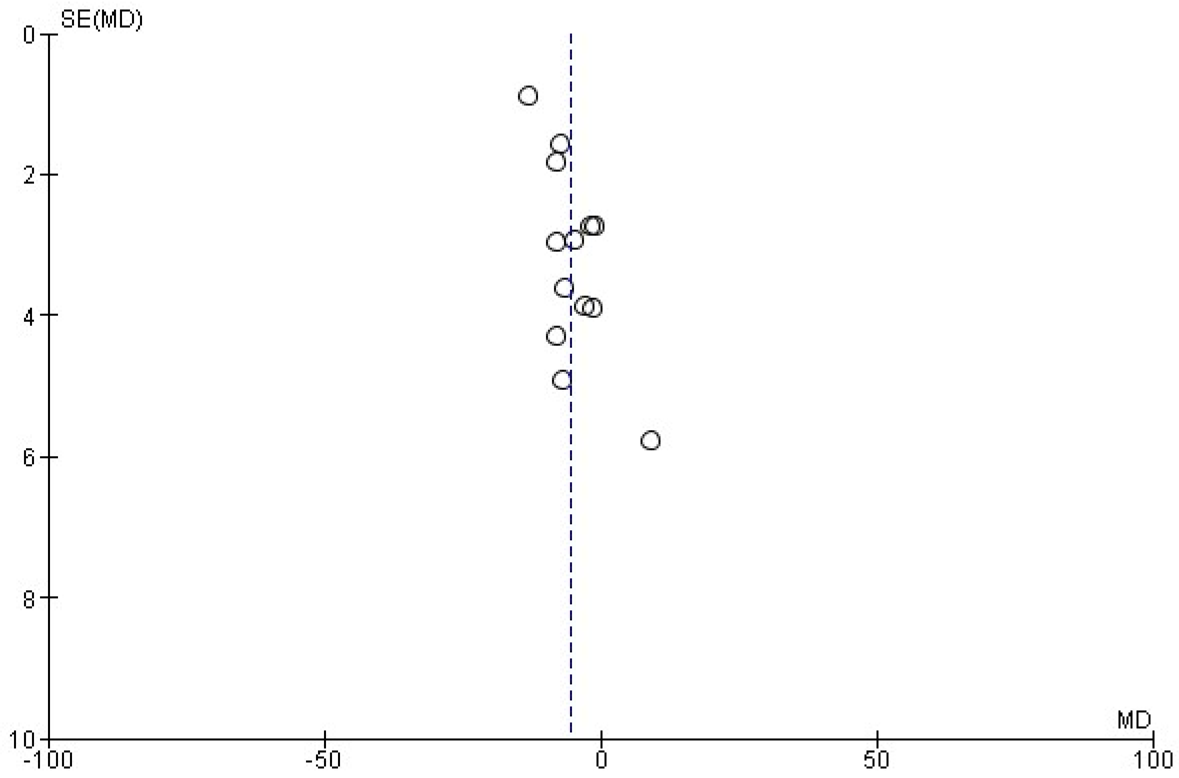

Figure 5: Forest plots of significant effects of RT on PD

5A: Forest plot of significant effects of RT on functional mobility-TUG

	Experimental			Control				Mean Difference
Study or Subgroup	Mean	SD	Total	Mean	SD	Total	Weight	IV, Random, 95% CI
De Lima et al., 2019	26.4	5.5	17	34.2	6.9	16	6.2%	-7.80 [-12.07, -3.53]
Leal et al., 2019	14.6	3.7	27	18.7	4.3	27	12.3%	-4.10 [-6.24, -1.96]
Vieira de Moraes Filho et al., 2020	7.4	2	25	10	2.7	15	14.4%	-2.60 [-4.18, -1.02]
Silva- Batista et al., 2016	8.74	1.85	13	10.13	2.38	13	14.2%	-1.39 [-3.03, 0.25]
Diddle et al., 2009	6.09	1.44	10	7.13	2.19	10	14.2%	-1.04 [-2.66, 0.58]
Strand et al., 2017	7.76	3.28	13	8.64	2.9	13	11.4%	-0.88 [-3.26, 1.50]
Demonceau et al., 2017	1.8	0.3	15	1.9	0.4	15	18.0%	-0.10 [-0.35, 0.15]
Cherup et al., 2019	8.75	5.49	18	7.96	3.58	18	9.3%	0.79 [-2.24, 3.82]
**Total (95% CI)**			**138**			**127**	**100.0%**	**-1.75 [-3.07, -0.44]**

Heterogeneity: Tau² = 2.48; Chi² = 37.74, df = 7 (P < 0.00001); I² = 81%

Test for overall effect: Z = 2.61 (P = 0.009)

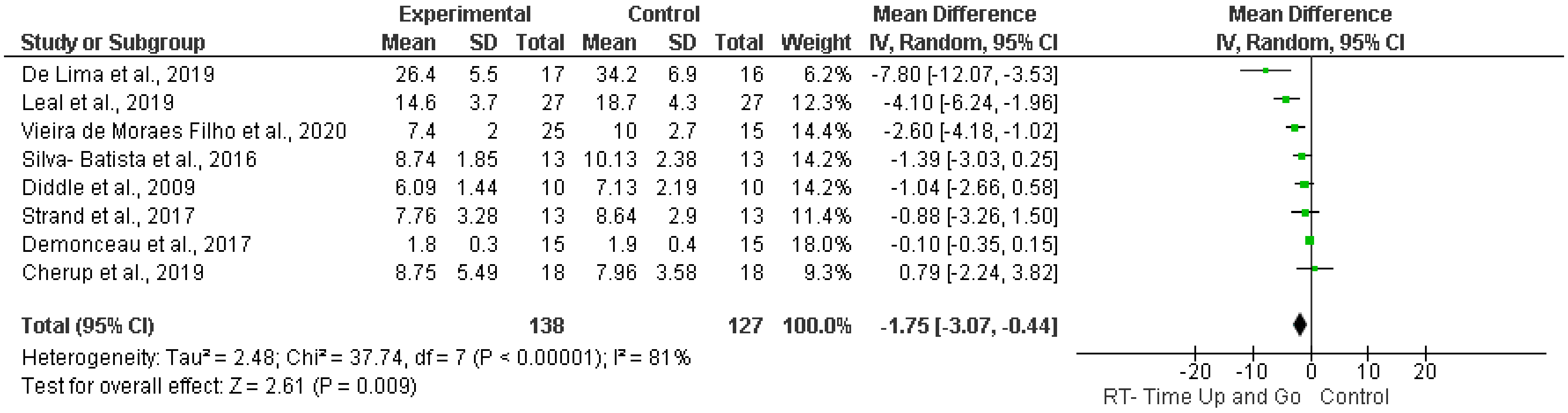

5B: Forest plot of significant effects of RT on QoL- PDQ-39

Study or Subgroup	Experimental Mean	Experimental SD	Experimental Total	Control Mean	Control SD	Control Total	Weight	Std. Mean Difference IV, Random, 95% CI
Santos et al., 2017	4.58	4.37	13	11.16	7.39	13	9.4%	-1.05 [-1.88, -0.22]
Ferreira et al, 2018	31.72	19.75	18	47.24	21.34	17	12.4%	-0.74 [-1.43, -0.05]
De Lima et al., 2019	30.2	16.8	17	40.6	15.6	16	12.0%	-0.63 [-1.33, 0.08]
Demonceau et al., 2017	19	7	15	24	12	15	11.4%	-0.50 [-1.22, 0.23]
Diddle et al., 2009	45.35	14.96	10	51.89	16.38	10	8.4%	-0.40 [-1.29, 0.49]
Strand et al., 2017	18.8	11.05	13	21.69	14.2	13	10.5%	-0.22 [-0.99, 0.55]
Silva- Batista et al., 2016	40.3	9.16	13	42.59	14.2	13	10.5%	-0.19 [-0.96, 0.59]
Morris et al., 2017	21	14	62	20	14	58	25.5%	0.07 [-0.29, 0.43]
**Total (95% CI)**			**161**			**155**	**100.0%**	**-0.38 [-0.67, -0.09]**

Heterogeneity: Tau² = 0.05; Chi² = 10.10, df = 7 (P = 0.18); I² = 31%
Test for overall effect: Z = 2.58 (P = 0.010)

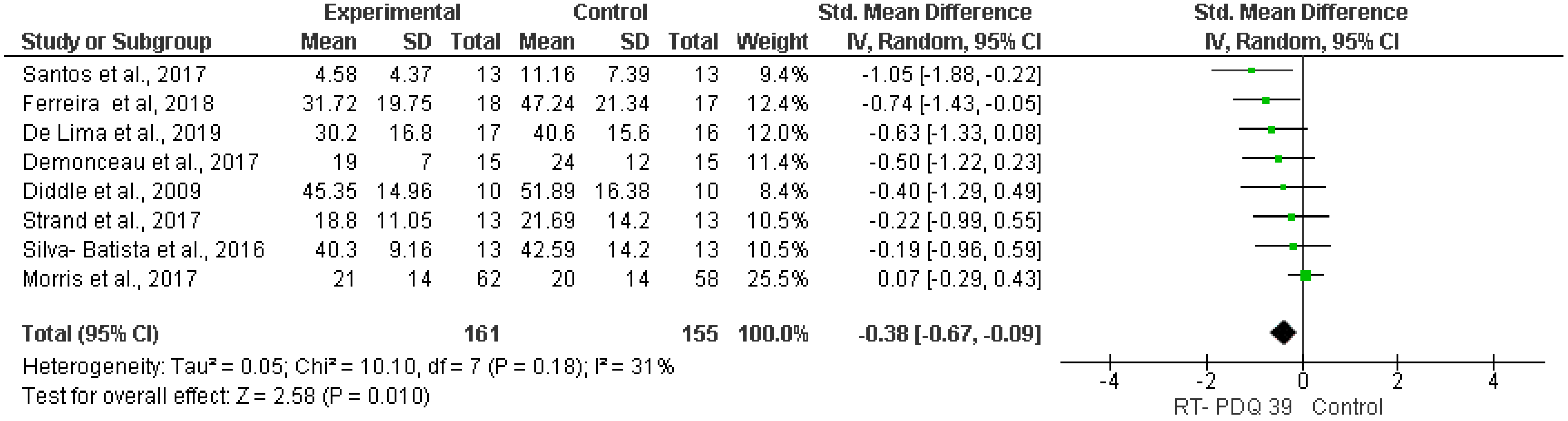

5C: Forest plot of significant effects of RT on lower limb strength- Leg Press

Study or Subgroup	Experimental Mean	Experimental SD	Experimental Total	Control Mean	Control SD	Control Total	Weight	Std. Mean Difference IV, Random, 95% CI
Silva- Batista et al., 2016	113	25.4	13	85.18	25.93	13	26.6%	1.05 [0.22, 1.88]
Scandalis et al., 2001	622	96	14	322	58	14	25.1%	3.67 [2.40, 4.95]
Cherup et al., 2019	177	13	18	122.2	11	18	25.1%	4.45 [3.18, 5.72]
Strand et al., 2017	620	83.2	13	181.6	81.7	13	23.2%	5.15 [3.45, 6.85]
**Total (95% CI)**			**58**			**58**	**100.0%**	**3.51 [1.50, 5.52]**

Heterogeneity: Tau² = 3.77; Chi² = 32.68, df = 3 (P < 0.00001); I² = 91%
Test for overall effect: Z = 3.42 (P = 0.0006)

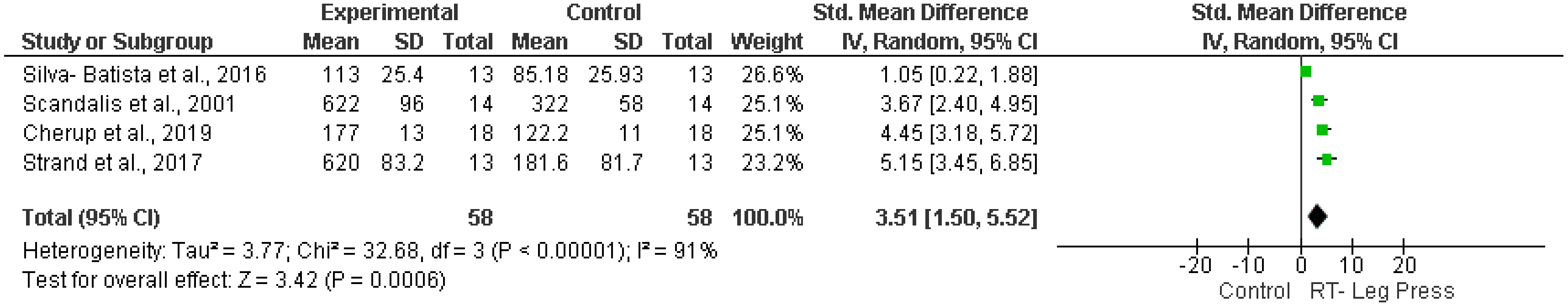

5D: Forest plot of significant effects of RT on lower limb strength- knee flexion

Study or Subgroup	Experimental Mean	Experimental SD	Experimental Total	Control Mean	Control SD	Control Total	Weight	Std. Mean Difference IV, Random, 95% CI
Demonceau et al., 2017	0.789	0.277	15	0.636	0.528	15	36.5%	0.35 [-0.37, 1.08]
Hass et al., 2012	41.5	14.59	11	26.4	14.59	11	31.7%	1.00 [0.10, 1.89]
Scandalis et al., 2001	258	71	14	151	44	14	31.8%	1.76 [0.87, 2.65]
**Total (95% CI)**			**40**			**40**	**100.0%**	**1.00 [0.18, 1.82]**

Heterogeneity: Tau² = 0.34; Chi² = 5.79, df = 2 (P = 0.06); I² = 65%
Test for overall effect: Z = 2.40 (P = 0.02)

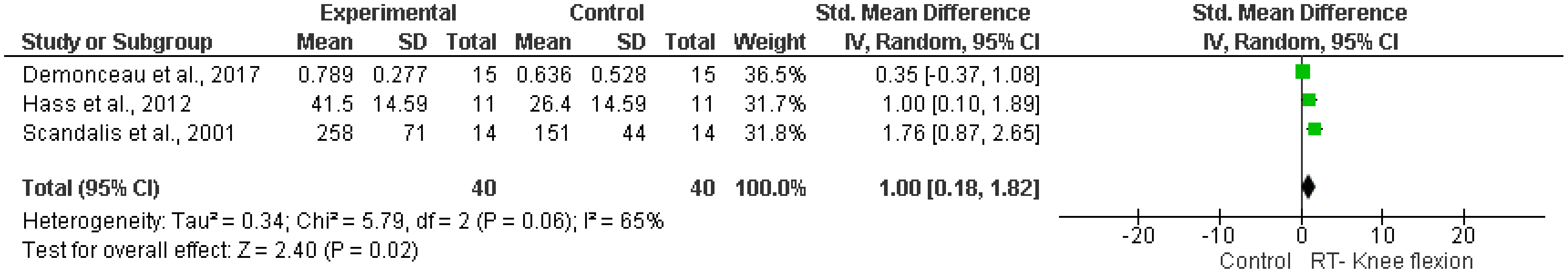

Figure 6: Forest plot of non-significant effects of rhythmic cueing on PD

6A: Forest plot of non-significant effects of rhythmic cueing on stride time

Study or Subgroup	Exercise RAS Mean	Exercise RAS SD	Exercise RAS Total	Control Mean	Control SD	Control Total	Weight	Std. Mean Difference IV, Random, 95% CI
Hausdorff et al., 2007	1.03	0.09	29	1.08	0.13	29	59.8%	-0.44 [-0.96, 0.08]
De Icco et al.,2015	1,246	263.4	8	1,250.5	317.2	8	16.9%	-0.01 [-0.99, 0.97]
De Bruin et al., 2010	1.06	0.07	11	1.06	0.13	11	23.3%	0.00 [-0.84, 0.84]
**Total (95% CI)**			**48**			**48**	**100.0%**	**-0.27 [-0.67, 0.14]**

Heterogeneity: Tau² = 0.00; Chi² = 1.08, df = 2 (P = 0.58); I² = 0%
Test for overall effect: Z = 1.29 (P = 0.20)

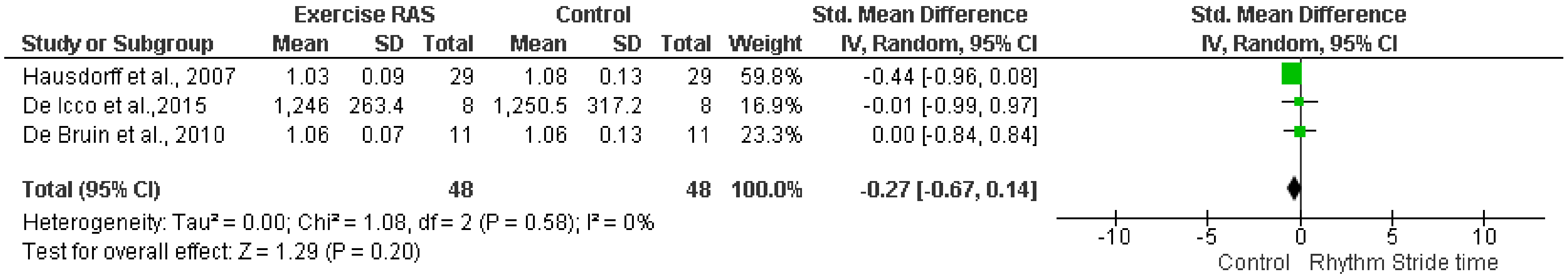

6B Forest plot of non-significant effects of rhythmic cueing on functional mobility-TUG

Study or Subgroup	Exercise RAS Mean	Exercise RAS SD	Exercise RAS Total	Control Mean	Control SD	Control Total	Weight	Mean Difference IV, Random, 95% CI
Kardival et al., 2011	9.4	3.46	8	15	6.34	8	42.3%	-5.60 [-10.60, -0.60]
Thaut et al., 2018	12.1	5.5	25	12	2.9	30	57.7%	0.10 [-2.29, 2.49]
**Total (95% CI)**			**33**			**38**	**100.0%**	**-2.31 [-7.83, 3.21]**

Heterogeneity: Tau² = 12.24; Chi² = 4.06, df = 1 (P = 0.04); I² = 75%
Test for overall effect: Z = 0.82 (P = 0.41)

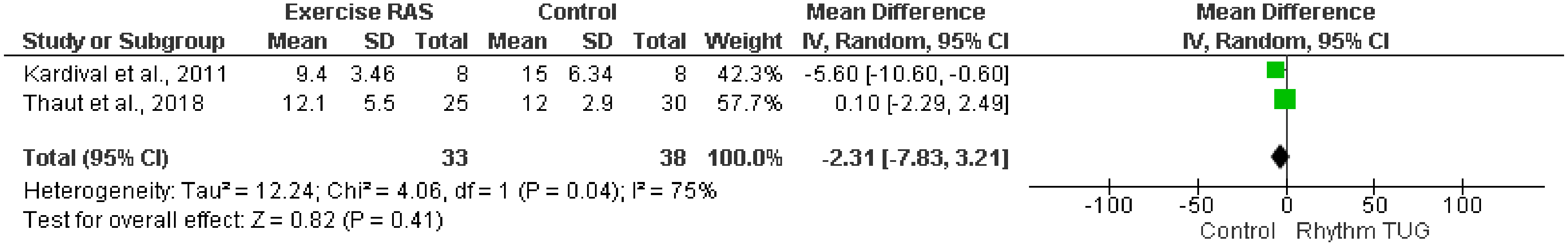

Figure 7: Forest plots non-significant effects of dance on PD

7A: Forest plot of non-significant effects of dance on gait velocity

Study or Subgroup	Experimental Mean	Experimental SD	Experimental Total	Control Mean	Control SD	Control Total	Weight	Std. Mean Difference IV, Random, 95% CI
Rawson et al., 2019	103.36	26.29	39	104.53	24.48	39	31.7%	-0.05 [-0.49, 0.40]
Dos Santos Delabary et al., 2020	1.1	0.24	12	1.08	0.14	12	9.7%	0.10 [-0.70, 0.90]
Hackney and Earhart, 2009 a waltz	1.13	0.2	17	1.09	0.41	17	13.8%	0.12 [-0.55, 0.79]
Hackney et al., 2007	0.88	0.12	9	0.86	0.12	9	7.3%	0.16 [-0.77, 1.08]
Hackney and Earhart, 2009 a Tango	1.19	0.18	14	1.09	0.41	17	12.3%	0.30 [-0.41, 1.01]
Harrison et al., 2020	1.42	0.29	11	1.33	0.27	11	8.8%	0.31 [-0.53, 1.15]
Hackney and Earhart, 2009 c Short Duration	1.1	0.2	12	1	0.2	12	9.4%	0.48 [-0.33, 1.30]
Solla et al., 2019	1.34	0.09	10	1.2	0.2	9	6.9%	0.88 [-0.07, 1.83]
**Total (95% CI)**			**124**			**126**	**100.0%**	**0.19 [-0.06, 0.44]**

Heterogeneity: Tau² = 0.00; Chi² = 3.85, df = 7 (P = 0.80); I² = 0%
Test for overall effect: Z = 1.52 (P = 0.13)

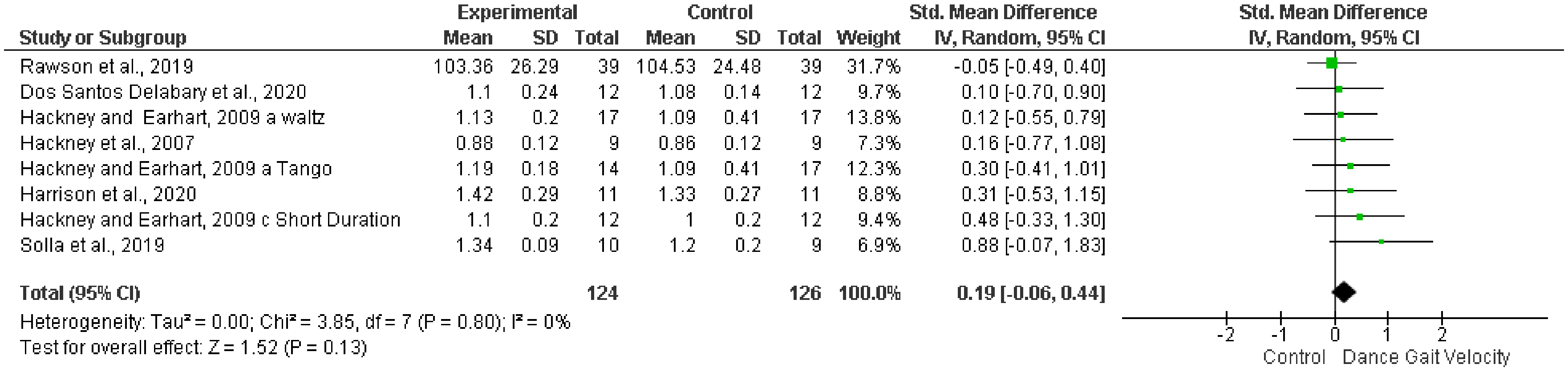

7B: Forest plot of non-significant effects of dance on QoL-PDQ-39

Study or Subgroup	Experimental Mean	Experimental SD	Experimental Total	Control Mean	Control SD	Control Total	Weight	Mean Difference IV, Random, 95% CI
Tilmann et al., 2020	49	27.9	20	66.4	9.3	20	7.5%	-17.40 [-30.29, -4.51]
Volpe et al.,2013	22.16	10.18	12	30.6	12.06	12	12.7%	-8.44 [-17.37, 0.49]
Kalyani et al., 2020	23.24	14.35	17	29.94	13.9	16	11.5%	-6.70 [-16.34, 2.94]
Rios Romenets et al., 2015	26.4	18.9	11	26.8	17.1	18	6.9%	-0.40 [-14.08, 13.28]
Westheimer et al.,2015	25.1	17.6	12	25.3	20.3	12	5.8%	-0.20 [-15.40, 15.00]
Harrison et al., 2020	23	15	11	23	13	11	8.7%	0.00 [-11.73, 11.73]
Kunkel, et al., 2017	19.9	12.5	30	18.3	10.8	15	16.5%	1.60 [-5.46, 8.66]
Poier et al., 2019	27.29	10.48	14	25.06	9.82	14	15.4%	2.23 [-5.29, 9.75]
Kalyani et al., 2019	19.45	12.45	17	16.82	10.14	16	15.0%	2.63 [-5.10, 10.36]
**Total (95% CI)**			**144**			**134**	**100.0%**	**-2.19 [-6.21, 1.84]**

Heterogeneity: Tau² = 12.50; Chi² = 12.17, df = 8 (P = 0.14); I² = 34%

Test for overall effect: Z = 1.07 (P = 0.29)

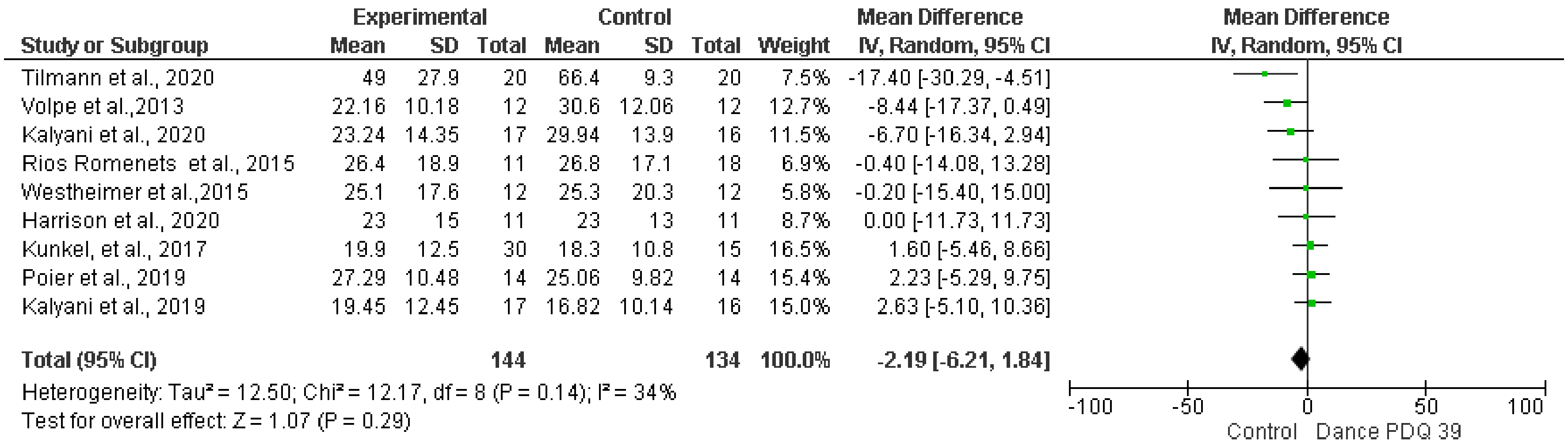

7C: Forest plot of non-significant effects of dance on cognition-MoCa

Study or Subgroup	Experimental Mean	Experimental SD	Experimental Total	Control Mean	Control SD	Control Total	Weight	Mean Difference IV, Random, 95% CI
Rios Romenets et al., 2015	27.4	2.1	11	27	2.4	18	68.3%	0.40 [-1.26, 2.06]
Rawson et al., 2019	18.8	11.55	39	18.33	11.8	39	7.0%	0.47 [-4.71, 5.65]
Solla et al., 2019	26.4	3.47	10	25.22	2.68	9	24.6%	1.18 [-1.59, 3.95]
**Total (95% CI)**			**60**			**66**	**100.0%**	**0.60 [-0.78, 1.97]**

Heterogeneity: Tau² = 0.00; Chi² = 0.23, df = 2 (P = 0.89); I² = 0%
Test for overall effect: Z = 0.85 (P = 0.40)

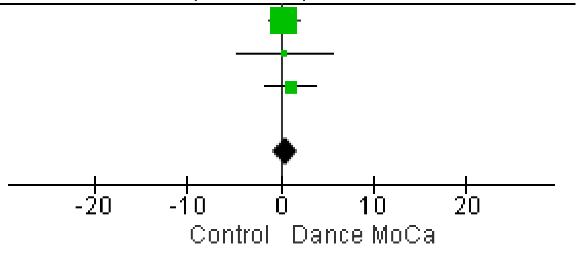

Figure 8 Forest plots non-significant effects of resistance training (RT) on Parkinson Disease
8A: Forest plot of non-significant effects of RT on gait velocity

Study or Subgroup	Experimental Mean	Experimental SD	Experimental Total	Control Mean	Control SD	Control Total	Weight	Std. Mean Difference IV, Random, 95% CI
Carvalho et al., 2015	6.2	3.1	8	8	3.1	8	12.7%	-0.55 [-1.55, 0.46]
Santos et al., 2017	0.85	0.15	13	0.87	0.15	13	17.1%	-0.13 [-0.90, 0.64]
Vieira de Moraes Filho et al., 2020	2	1.5	25	1.7	1.16	15	20.1%	0.21 [-0.43, 0.85]
Demonceau et al., 2017	1.22	0.1	15	1.16	0.2	15	18.2%	0.37 [-0.35, 1.09]
Diddle et al., 2009	1.97	0.38	10	1.74	0.33	10	14.4%	0.62 [-0.28, 1.52]
De Lima et al., 2019	1.25	0.34	17	0.91	0.19	16	17.6%	1.19 [0.45, 1.94]
**Total (95% CI)**			**88**			**77**	**100.0%**	**0.32 [-0.13, 0.77]**

Heterogeneity: Tau² = 0.16; Chi² = 9.97, df = 5 (P = 0.08); I² = 50%
Test for overall effect: Z = 1.37 (P = 0.17)

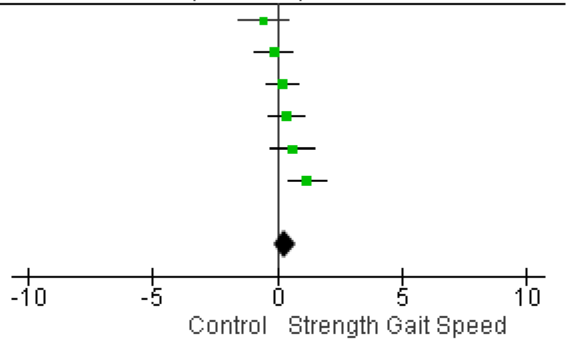

8B: Forest plot of non-significant effects of RT on stride length

Study or Subgroup	Experimental Mean	Experimental SD	Experimental Total	Control Mean	Control SD	Control Total	Weight	Mean Difference IV, Random, 95% CI
Demonceau et al., 2017	1.27	0.14	15	1.24	0.19	15	76.4%	0.03 [-0.09, 0.15]
Scandalis et al., 2001	0.95	0.3	14	0.83	0.28	14	23.6%	0.12 [-0.09, 0.33]
**Total (95% CI)**			**29**			**29**	**100.0%**	**0.05 [-0.05, 0.16]**

Heterogeneity: $Tau^2 = 0.00$; $Chi^2 = 0.51$, df = 1 (P = 0.47); $I^2 = 0\%$
Test for overall effect: Z = 0.96 (P = 0.34)

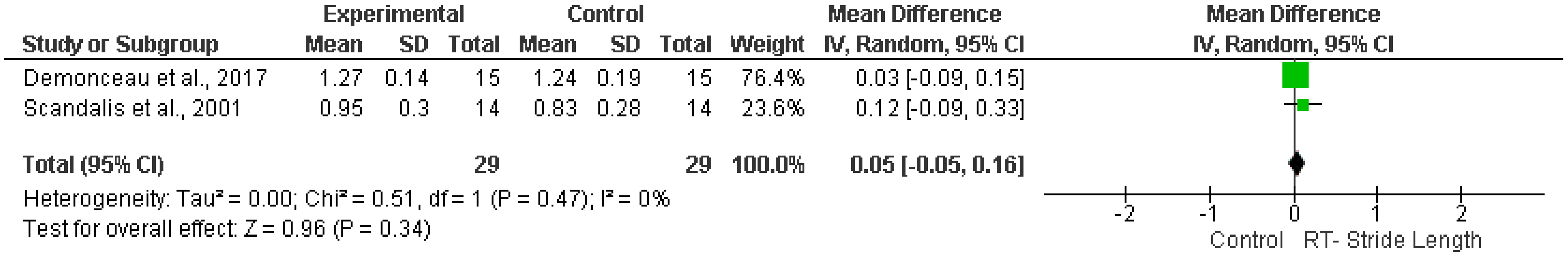

8C: Forest plot of non-significant effects of RT on motor symptoms-UPDRS-III

Study or Subgroup	Experimental Mean	Experimental SD	Experimental Total	Control Mean	Control SD	Control Total	Weight	Mean Difference IV, Random, 95% CI
Carvalho et al., 2015	30.5	8.3	8	42.1	10.2	8	9.4%	-11.60 [-20.71, -2.49]
Strand et al., 2017	24.46	10.81	13	28.85	12.91	13	9.3%	-4.39 [-13.54, 4.76]
Silva- Batista et al., 2016	43	12.86	13	45.36	7.84	13	11.7%	-2.36 [-10.55, 5.83]
Morris et al., 2017	28	14	62	30	13	58	33.0%	-2.00 [-6.83, 2.83]
Diddle et al., 2009	9	4.6	8	10.75	5.97	8	28.4%	-1.75 [-6.97, 3.47]
Ferreira et al, 2018	33.6	20.2	18	31.2	6.3	17	8.2%	2.40 [-7.40, 12.20]
**Total (95% CI)**			**122**			**117**	**100.0%**	**-2.74 [-5.55, 0.07]**

Heterogeneity: $Tau^2 = 0.13$; $Chi^2 = 5.05$, df = 5 (P = 0.41); $I^2 = 1\%$
Test for overall effect: Z = 1.91 (P = 0.06)

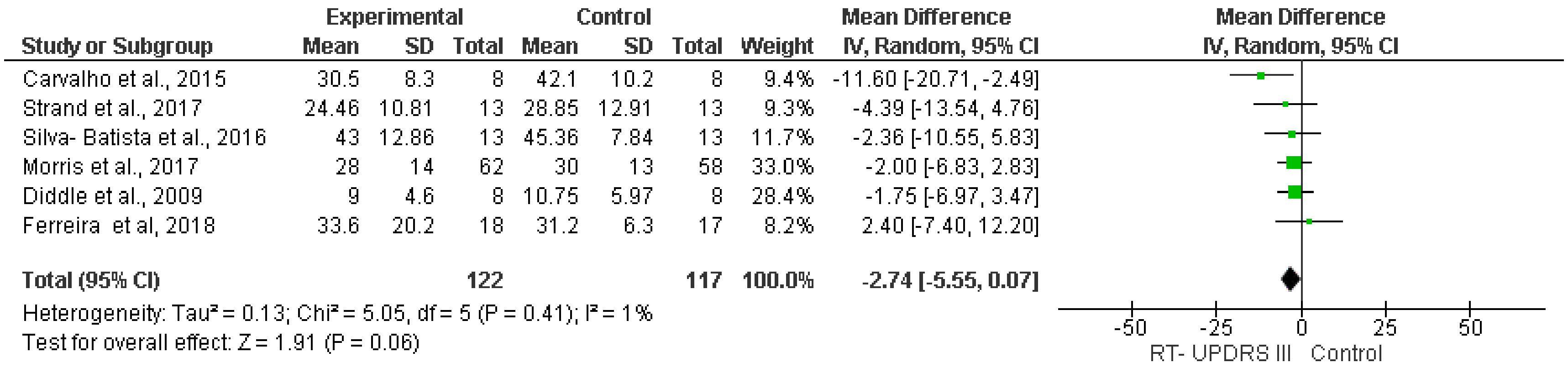

8D: Forest plot of non-significant effects of RT on lower limb strength-knee extension

Study or Subgroup	Experimental Mean	Experimental SD	Experimental Total	Control Mean	Control SD	Control Total	Weight	Std. Mean Difference IV, Random, 95% CI
Scandalis et al., 2001	451	74	14	207	0	14		Not estimable
Vieira de Moraes Filho et al., 2020	147.1	38.5	25	135.8	43.3	15	20.7%	0.27 [-0.37, 0.92]
Diddle et al., 2009	330.1	92.54	10	269.1	85.69	10	19.9%	0.66 [-0.25, 1.56]
Diddle et al., 2006	90.74	31.03	10	69.94	23.98	10	19.9%	0.72 [-0.19, 1.63]
Hass et al., 2012	75.9	28.19	11	43	28.19	11	19.8%	1.12 [0.21, 2.04]
Silva Batista et al., 2017	108	28	11	67	29	11	19.7%	1.38 [0.43, 2.33]
Demonceau et al., 2017	1,736	0.453	15	1,488	0.528	15	0.0%	490.51 [357.34, 623.68]
**Total (95% CI)**			**96**			**86**	**100.0%**	**0.88 [-0.54, 2.30]**

Heterogeneity: Tau² = 2.41; Chi² = 56.45, df = 5 (P < 0.00001); I² = 91%
Test for overall effect: Z = 1.22 (P = 0.22)

Std. Mean Difference
IV, Random, 95% CI

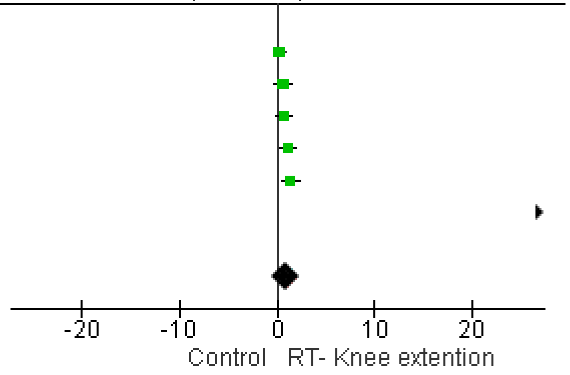

Table 4: GRADE analysis outcomes

		Summary of findings table according to GRADE analysis			Methodological design start point	Evaluation components to lower quality				
No	Meta-analysis No	Outcome	Prevalence/risk ratio (95% CI)/Counts	Quality of evidence (GRADE)		Risk of bias	Inconsistencey of results	Indirectness	Imprecision	Publication bias
1	Supplement Fig. 3 A	Rhythm velocity/speed	0.54 [0.21, 0.87]%	Moderate ⊕⊕⊕◯	RCTs and CTs: Moderate quality	The included studies display 69% low risk of bias. **No downgrade**	Even though we used a random effect model meta-analysis, we consider heterogeneity as an index of inconsistency, when this was >75% (considerable heterogeneity). **I^2=46%.** **No downgrade**	Most of the studies do display as a primary aim, very similar to the systematic review aim. Therefore, the available evidence is applicable to our research question. **No downgrade**	We considered optimal information size of n=110 participants (power calculation by Rawson et. Al. 2019). **Sample size (n=269) No downgrade**	Due to <10 entries in most meta-analyses were imported, funnel plots were not produced. We considered study design of the studies included in each meta-analysis and small sample sizes (<110) for downgrading. **RCTs and CTs included. Sample size >110 No downgrade**

2	Supplement Fig. 3 B	Rhythm stride length	0.09 [0.03, 0.15] %	Moderate ⊕⊕⊕◯	RCTs and CTs: Moderate quality	The included studies display 69% low risk of bias. **No downgrade**	Even though we used a random effect model meta-analysis, we consider heterogeneity as an index of inconsistency, when this was >75% (considerable heterogeneity). **I^2=46% No downgrade**	Most of the studies do display as a primary aim, very similar to the systematic review aim. Therefore, the available evidence is applicable to our research question. **No downgrade**	We considered optimal information size of n=110 participants (power calculation by Rawson et. Al. 2019). **Sample size (n=296) No Downgrade**	Due to <10 entries in most meta-analyses were imported, funnel plots were not produced. We considered study design of the studies included in each meta-analysis and small sample sizes (<110) for downgrading. **RCTs and CTs included. Sample size >110 No downgrade**
3	Supplement Fig.6A	Rhythm stride time	-0.27 [-0.67, 0.14] %	Very low ⊕◯◯◯	RCTs and CTs: Moderate quality	The included studies display 47% low risk of bias **Downgrade 1 level.**	Even though we used a random effect model meta-analysis, we consider heterogeneity as an index of inconsistency, when this was >75% (considerable heterogeneity) **I^2=0% No**	Most of the studies do display as a primary aim, very similar to the systematic review aim. Therefore, the available evidence is applicable to our research question. **No downgrade**	We considered optimal information size of n=110 participants (power calculation by Rawson et. Al. 2019). **Sample size (n=96) Downgrade**	Due to <10 entries in most meta-analyses were imported, funnel plots were not produced. We considered study design of the studies included in each meta-analysis and small sample sizes (<110) for downgrading. **RCTs and CTs included. Sample size <110 Downgrade 1 level**

							**downgrade**		**1 level**	
4	Supplement Fig. 6B	Rhythm TUG	-2.32(-7.83, 3.21) %	Low ⊕⊕◯◯	RCTs: High quality	The included studies display 75% low risk of bias **No downgrade**	Even though we used a random effect model meta-analysis, we consider heterogeneity as an index of inconsistency, when this was >75% (considerable heterogeneity) **I^2=75%** **No downgrade**	Most of the studies do display as a primary aim, very similar to the systematic review aim. Therefore, the available evidence is applicable to our research question. **No downgrade**	We considered optimal information size of n=110 participants (power calculation by Rawson et. Al. 2019). **Sample size (n=71) Downgrade 1 level**	Due to <10 entries in most meta-analyses were imported, funnel plots were not produced. We considered study design of the studies included in each meta-analysis and small sample sizes (<110) for downgrading. **RCTs included Sample size <110 Downgrade 1 level**
5	Supplement Fig. 3C	Rhytym UPDRS-III	-5.60 [-9.48, -1.72] %	Low ⊕⊕◯◯	RCTs: High quality	The included studies display >83% low risk of bias. **No downgrade**	Even though we used a random effect model meta-analysis, we consider heterogeneity as an index of inconsistency, when this was >75% (considerable heterogeneity) **I^2=7%** **No downgrade**	Most of the studies do display as a primary aim, very similar to the systematic review aim. Therefore, the available evidence is applicable to our research question. **No downgrade**	We considered optimal information size of n=110 participants (power calculation by Rawson et. Al. 2019). **Sample size (n=102) Downgrade 1 level**	Due to <10 entries in most meta-analyses were imported, funnel plots were not produced. We considered study design of the studies included in each meta-analysis and small sample sizes (<110) for downgrading. **RCT included Sample size <110 Downgrade 1 level**

6	Supplement Fig.7A	Dance Gait velocity/speed	0.19 [-0.06, 0.44]	Moderate ⊕⊕⊕◯	RCTs and CTs: Moderate quality	The included studies display >80% low risk of bias. **No downgrade**	Even though we used a random effect model meta-analysis, we consider heterogeneity as an index of inconsistency, when this was >75% (considerable heterogeneity) **I^2=0% No downgrade**	Most of the studies do display as a primary aim, very similar to the systematic review aim. Therefore, the available evidence is applicable to our research question. **No downgrade**	We considered optimal information size of n=110 participants (power calculation by Rawson et. Al. 2019). **Sample size (n=250) No downgrade**	Due to <10 entries in most meta-analyses were imported, funnel plots were not produced. We considered study design of the studies included in each meta-analysis and small sample sizes (<110) for downgrading. **RCTs and CTs included Sample size >110 No downgrade**
7	Supplement Fig.4A	Dance Gait Stride length	0.07 [0.00, 0.15]	Moderate ⊕⊕⊕◯	RCTs and CTs: Moderate quality	The included studies display >80% low risk of bias. **No downgrade**	Even though we used a random effect model meta-analysis, we consider heterogeneity as an index of inconsistency, when this was >75% (considerable heterogeneity) **I^2=0% No downgrade**	Most of the studies do display as a primary aim, very similar to the systematic review aim. Therefore, the available evidence is applicable to our research question.	We considered optimal information size of n=110 participants (power calculation by Rawson et. Al. 2019). **Sample size (n=140) No downgrade**	Due to <10 entries in most meta-analyses were imported, funnel plots were not produced. We considered study design of the studies included in each meta-analysis and small sample sizes (<110) for downgrading. **RCTs and CTs included Sample size >110 No downgrade**

								**No downgrade**		
8	Supplement Fig4B	Dance TUG	-1.26 [-1.77, -0.75]	Moderate ⊕⊕⊕◯	RCTs and CTs: Moderate quality	The included studies display >72% low risk of bias. **No downgrade**	Even though we used a random effect model meta-analysis, we consider heterogeneity as an index of inconsistency, when this was >75% (considerable heterogeneity) **I^2=0%** **No downgrade**	Most of the studies do display as a primary aim, very similar to the systematic review aim. Therefore, the available evidence is applicable to our research question. **No downgrade**	We considered optimal information size of n=110 participants (power calculation by Rawson et. Al. 2019). **Sample size (n=320)** **No downgrade**	Due to <10 entries in most meta-analyses were imported, funnel plots were not produced. We considered study design of the studies included in each meta-analysis and small sample sizes (<110) for downgrading. **RCTs and CTs included** **Sample size >110** **No downgrade**

9	Supplement Fig.4C	Dance UPDRS-III	-5.38 [-8.44, -2.32]	Very low ⊕◯◯◯	CTs: Low quality	The included studies display >91% low risk of bias. **No downgrade**	Even though we used a random effect model meta-analysis, we consider heterogeneity as an index of inconsistency, when this was >75% (considerable heterogeneity) **I^2=79%** **Downgrade 1 level**	Most of the studies do display as a primary aim, very similar to the systematic review aim. Therefore, the available evidence is applicable to our research question. **No downgrade**	We considered optimal information size of n=110 participants (power calculation by Rawson et. Al. 2019). **Sample size (n=406)** **No downgrade**	Due to <10 entries in most meta-analyses were imported, funnel plots were not produced. We considered study design of the studies included in each meta-analysis and small sample sizes (<110) for downgrading. **CTs included** **Sample size >110** **No downgrade**
10	Supplement Fig.7B	Dance PDQ-39	-2.19 [-6.21, 1.84]	Moderate ⊕⊕⊕◯	RCTs and CTs: Moderate quality	The included studies display >69% low risk of bias. **No downgrade**	Even though we used a random effect model meta-analysis, we consider heterogeneity as an index of inconsistency, when this was >75% (considerable heterogeneity) **I^2=34%** **No downgrade**	Most of the studies do display as a primary aim, very similar to the systematic review aim. Therefore, the available evidence is applicable to our research question. **No downgrade**	We considered optimal information size of n=110 participants (power calculation by Rawson et. Al. 2019). **Sample size (n=278)** **No**	Due to <10 entries in most meta-analyses were imported, funnel plots were not produced. We considered study design of the studies included in each meta-analysis and small sample sizes (<110) for downgrading. **RCTs and CTs included** **Sample size >110** **No downgrade**

									**downgrade**	
11	Supplement Fig.7C	Dance MoCa	0.60 [-0.78, 1.97]	Moderate ⊕⊕⊕◯	RCTs and CTs: Moderate quality	The included studies display >78% low risk of bias.<br><br>**No downgrade**	Even though we used a random effect model meta-analysis, we consider heterogeneity as an index of inconsistency, when this was >75% (considerable heterogeneity) **I^2=0%** **No Downgrade**	Most of the studies do display as a primary aim, very similar to the systematic review aim. Therefore, the available evidence is applicable to our research question. **No downgrade**	We considered optimal information size of n=110 participants (power calculation by Rawson et. Al. 2019). **Sample size (n=126)** **No downgrade**	Due to <10 entries in most meta-analyses were imported, funnel plots were not produced. We considered study design of the studies included in each meta-analysis and small sample sizes (<110) for downgrading. **RCTs and CTs included Sample size >110 No downgrade**

12	Supplement Fig.8A	RT- Gait velocity/speed	0.32 [-0.13, 0.77] %	Moderate ⊕⊕⊕◯	RCTs and CTs: Moderate quality	The included studies display 78% low risk of bias. **No downgrade**	Even though we used a random effect model meta-analysis, we consider heterogeneity as an index of inconsistency, when this was >75% (considerable heterogeneity) **I^2=50%** **No downgrade**	Most of the studies do display as a primary aim, very similar to the systematic review aim. Therefore, the available evidence is applicable to our research question. **No downgrade**	We considered optimal information size of n=110 participants (power calculation by Rawson et. Al. 2019). **Sample size (n=165) No downgrade**	Due to <10 entries in most meta-analyses were imported, funnel plots were not produced. We considered study design of the studies included in each meta-analysis and small sample sizes (<110) for downgrading. **RCTs and CTs included Sample size >110 No downgrade**
13	Supplement Fig.8B	RT- Stride length	0.05 [-0.05, 0.16]	Very low ⊕◯◯◯	CTs: Low	The included studies display 57% low risk of bias. **No downgrade**	Even though we used a random effect model meta-analysis, we consider heterogeneity as an index of inconsistency, when this was >75% (considerable heterogeneity) **I^2=0%** **No downgrade**	Most of the studies do display as a primary aim, very similar to the systematic review aim. Therefore, the available evidence is applicable to our research question. **No**	We considered optimal information size of n=110 participants (power calculation by Rawson et. Al. 2019). **Sample size (n=58) Downgrade 1 level**	Due to <10 entries in most meta-analyses were imported, funnel plots were not produced. We considered study design of the studies included in each meta-analysis and small sample sizes (<110) for downgrading. **CTs included Sample size <110 Downgrade 1 level**

								**downgrade**		
14	Supplement Fig.5A	RT- TUG	-1.75 [-3.10, -0.40]	Low ⊕⊕◯◯	RCTs and CTs: Moderate quality	The included studies display >80% low risk of bias. **No downgrade**	Even though we used a random effect model meta-analysis, we consider heterogeneity as an index of inconsistency, when this was >75% (considerable heterogeneity) **I^2=81% Downgrade 1 level**	Most of the studies do display as a primary aim, very similar to the systematic review aim. Therefore, the available evidence is applicable to our research question. **No downgrade**	We considered optimal information size of n=110 participants (power calculation by Rawson et. Al. 2019). **Sample size (n=265) No downgrade**	Due to <10 entries in most meta-analyses were imported, funnel plots were not produced. We considered study design of the studies included in each meta-analysis and small sample sizes (<110) for downgrading. **RCTs and CTs included Sample size >110 No downgrade**
15	Supplement Fig.8C	RT-UPDRS-III	-2.74 [-5.55, 0.07]	Moderate ⊕⊕⊕◯	RCT and CTs: Moderate quality	The included studies display 81% low risk of bias. **No downgrade**	Even though we used a random effect model meta-analysis, we consider heterogeneity as an index of inconsistency, when this was >75% (considerable heterogeneity) **I^2=1%**	Most of the studies do display as a primary aim, very similar to the systematic review aim. Therefore, the available evidence is applicable	We considered optimal information size of n=110 participants (power calculation by Rawson et. Al. 2019). **Sample size (**	Due to <10 entries in most meta-analyses were imported, funnel plots were not produced. We considered study design of the studies included in each meta-analysis and small sample sizes (<110) for downgrading. **RCTs and CTs included Sample size >110 No downgrade**

							**No downgrade**	to our research question. **No downgrade**	n=239) **No downgrade**	
16	Supplement Fig.5B	RT- PDQ-39	-0.38 [-0.67, -0.09]	Moderate ⊕⊕⊕◯	RCTs and CTs: Moderate quality	The included studies display 86% low risk of bias. **No downgrade**	Even though we used a random effect model meta-analysis, we consider heterogeneity as an index of inconsistency, when this was >75% (considerable heterogeneity) **I^2=31% No downgrade**	Most of the studies do display as a primary aim, very similar to the systematic review aim. Therefore, the available evidence is applicable to our research question. **No downgrade**	We considered optimal information size of n=110 participants (power calculation by Rawson et. Al. 2019). **Sample size (n=316) No downgrade**	Due to <10 entries in most meta-analyses were imported, funnel plots were not produced. We considered study design of the studies included in each meta-analysis and small sample sizes (<110) for downgrading. **RCTs and CTs included Sample size >110 No downgrade**

17	Supplement Fig.5C	RT- Leg Press	3.51 [1.50, 5.52]	Low ⊕⊕◯◯	RCT and CTS: Moderate quality	The included studies display 83% low risk of bias. **No downgrade**	Even though we used a random effect model meta-analysis, we consider heterogeneity as an index of inconsistency, when this was >75% (considerable heterogeneity) **I^2=91% Downgrade 1 level**	Most of the studies do display as a primary aim, very similar to the systematic review aim. Therefore, the available evidence is applicable to our research question. **No downgrade**	We considered optimal information size of n=110 participants (power calculation by Rawson et. Al. 2019). **Sample size (n=116) No downgrade**	Due to <10 entries in most meta-analyses were imported, funnel plots were not produced. We considered study design of the studies included in each meta-analysis and small sample sizes (<110) for downgrading. **RCTs and CTs included Sample size >110 No downgrade**
18	Supplement Fig.5D	RT- Knee Flexion	1.00 [0.18, 1.82]	Very low ⊕◯◯◯	RCTs and CTs: Moderate quality	The included studies display 50% low risk of bias. **No downgrade**	Even though we used a random effect model meta-analysis, we consider heterogeneity as an index of inconsistency, when this was >75% (considerable heterogeneity) **I^2=65% No downgrade**	Most of the studies do display as a primary aim, very similar to the systematic review aim. Therefore, the available evidence is applicable to our research question. **No downgrade**	We considered optimal information size of n=110 participants (power calculation by Rawson et. Al. 2019). **Sample size (n=80) Downgrade 1 level**	Due to <10 entries in most meta-analyses were imported, funnel plots were not produced. We considered study design of the studies included in each meta-analysis and small sample sizes (<110) for downgrading. **RCTs and CTs included. Sample size <110 Downgrade 1 level**

19	Supplement Fig.8D	RT-Knee extension	1.32 [-0.09, 2.73]	Low ⊕⊕◯◯	RCTs and CTs: Moderate quality	The included studies display 60% low risk of bias. **No downgrade**	Even though we used a random effect model meta-analysis, we consider heterogeneity as an index of inconsistency, when this was >75% (considerable heterogeneity) **I^2=91% Downgrade 1 level**	Most of the studies do display as a primary aim, very similar to the systematic review aim. Therefore, the available evidence is applicable to our research question. **No downgrade**	We considered optimal information size of n=110 participants (power calculation by Rawson et. Al. 2019). **Sample size (n=182) No downgrade**	Due to <10 entries in most meta-analyses were imported, funnel plots were not produced. We considered study design of the studies included in each meta-analysis and small sample sizes (<110) for downgrading. **RCTs and CTs included Sample size >110 No downgrade**

PRISMA **CHECKLIST**

PRISMA 2020 Main Checklist

Topic	No.	Item	Location where item is reported
**TITLE**			
**Title**	1	Identify the report as a systematic review.	Page 2
**ABSTRACT**			
**Abstract**	2	See the PRISMA 2020 for Abstracts checklist	
**INTRODUCTION**			

Topic	No.	Item	Location where item is reported
**Rationale**	3	Describe the rationale for the review in the context of existing knowledge.	Section 1 pages 2-3
**Objectives**	4	Provide an explicit statement of the objective(s) or question(s) the review addresses.	Section 1 page 3
**METHODS**			
**Eligibility criteria**	5	Specify the inclusion and exclusion criteria for the review and how studies were grouped for the syntheses.	Section 2 pages 3-4
**Information sources**	6	Specify all databases, registers, websites, organisations, reference lists and other sources searched or consulted to identify studies. Specify the date when each source was last searched or consulted.	Section 2 page 4
**Search strategy**	7	Present the full search strategies for all databases, registers and websites, including any filters and limits used.	Section 2 page 4
**Selection process**	8	Specify the methods used to decide whether a study met the inclusion criteria of the review, including how many reviewers screened each record and each report retrieved, whether they worked independently, and if applicable, details of automation tools used in the process.	Section 2 page 4
**Data collection process**	9	Specify the methods used to collect data from reports, including how many reviewers collected data from each report, whether they worked independently, any processes for obtaining or confirming data from study investigators, and if applicable, details of automation tools used in the process.	Section 2 page 4
**Data items**	10a	List and define all outcomes for which data were sought. Specify whether all results that were compatible with each outcome domain in each study were sought (e.g. for all measures, time points, analyses), and if not, the methods used to decide which results to collect.	Section 2 page 4
	10b	List and define all other variables for which data were sought (e.g. participant and intervention characteristics, funding sources). Describe any assumptions made about any missing or unclear information.	Section 2 pages 4-5
**Study risk of bias assessment**	11	Specify the methods used to assess risk of bias in the included studies, including details of the tool(s) used, how many reviewers assessed each study and whether they worked independently, and if applicable, details of automation tools used in the process.	Section 2 page 4
**Effect measures**	12	Specify for each outcome the effect measure(s) (e.g. risk ratio, mean difference) used in the synthesis or presentation of results.	Section 2 pages 4-5
**Synthesis methods**	13a	Describe the processes used to decide which studies were eligible for each synthesis (e.g. tabulating the study intervention characteristics and comparing against the planned groups for each synthesis (item 5)).	Section 2 pages 4-5
	13b	Describe any methods required to prepare the data for presentation or synthesis, such as handling of missing summary statistics, or data conversions.	Section 2 pages 4-5

Topic	No.	Item	Location where item is reported
	13c	Describe any methods used to tabulate or visually display results of individual studies and syntheses.	Section 2 pages 4-5
	13d	Describe any methods used to synthesize results and provide a rationale for the choice(s). If meta-analysis was performed, describe the model(s), method(s) to identify the presence and extent of statistical heterogeneity, and software package(s) used.	Section 2 pages 4-5
	13e	Describe any methods used to explore possible causes of heterogeneity among study results (e.g. subgroup analysis, meta-regression).	Section 2 pages 4-5
	13f	Describe any sensitivity analyses conducted to assess robustness of the synthesized results.	Section 2 pages 4-5
**Reporting bias assessment**	14	Describe any methods used to assess risk of bias due to missing results in a synthesis (arising from reporting biases).	Section 2 pages 4-5
**Certainty assessment**	15	Describe any methods used to assess certainty (or confidence) in the body of evidence for an outcome.	Section 2 page 8
**RESULTS**			
**Study selection**	16a	Describe the results of the search and selection process, from the number of records identified in the search to the number of studies included in the review, ideally using a flow diagram.	Section 3 pages 5-6
	16b	Cite studies that might appear to meet the inclusion criteria, but which were excluded, and explain why they were excluded.	Section 3 page 6
**Study characteristics**	17	Cite each included study and present its characteristics.	Supplement
**Risk of bias in studies**	18	Present assessments of risk of bias for each included study.	Section 3 pages 6-7
**Results of individual studies**	19	For all outcomes, present, for each study: (a) summary statistics for each group (where appropriate) and (b) an effect estimate and its precision (e.g. confidence/credible interval), ideally using structured tables or plots.	Section 3 pages 7-8
**Results of syntheses**	20a	For each synthesis, briefly summarise the characteristics and risk of bias among contributing studies.	Section 3 Pages 6-7
	20b	Present results of all statistical syntheses conducted. If meta-analysis was done, present for each the summary estimate and its precision (e.g. confidence/credible interval) and measures of statistical heterogeneity. If comparing groups, describe the direction of the effect.	Section 3 Pages 6-7
	20c	Present results of all investigations of possible causes of heterogeneity among study results.	Section 3 Pages 6-7
	20d	Present results of all sensitivity analyses conducted to assess the robustness of the synthesized results.	Section 3 Pages 6-7
**Reporting biases**	21	Present assessments of risk of bias due to missing results (arising from reporting biases) for each synthesis assessed.	NA

Topic	No.	Item	Location where item is reported
**Certainty of evidence**	22	Present assessments of certainty (or confidence) in the body of evidence for each outcome assessed.	Section 3 page 8
**DISCUSSION**			
**Discussion**	23a	Provide a general interpretation of the results in the context of other evidence.	Section 4 page 8
	23b	Discuss any limitations of the evidence included in the review.	Section 4 pages 8-9
	23c	Discuss any limitations of the review processes used.	Section 4 page 10
	23d	Discuss implications of the results for practice, policy, and future research.	Section 4 page 10
**OTHER INFORMATION**			
**Registration and protocol**	24a	Provide registration information for the review, including register name and registration number, or state that the review was not registered.	Section 2 page 3
	24b	Indicate where the review protocol can be accessed, or state that a protocol was not prepared.	Section 2 page 3
	24c	Describe and explain any amendments to information provided at registration or in the protocol.	Section 2 page 3
**Support**	25	Describe sources of financial or non-financial support for the review, and the role of the funders or sponsors in the review.	Section 4 page 11
**Competing interests**	26	Declare any competing interests of review authors.	Section 4 page 11
**Availability of data, code and other materials**	27	Report which of the following are publicly available and where they can be found: template data collection forms; data extracted from included studies; data used for all analyses; analytic code; any other materials used in the review.	Section 4 page 11

PRIMSA Abstract Checklist

Topic	No.	Item	Reported?
**TITLE**			
**Title**	1	Identify the report as a systematic review.	Yes
**BACKGROUND**			

Topic	No.	Item	Reported?
**Objectives**	2	Provide an explicit statement of the main objective(s) or question(s) the review addresses.	Yes
**METHODS**			
**Eligibility criteria**	3	Specify the inclusion and exclusion criteria for the review.	Yes
**Information sources**	4	Specify the information sources (e.g. databases, registers) used to identify studies and the date when each was last searched.	Yes
**Risk of bias**	5	Specify the methods used to assess risk of bias in the included studies.	Yes
**Synthesis of results**	6	Specify the methods used to present and synthesize results.	Yes
**RESULTS**			
**Included studies**	7	Give the total number of included studies and participants and summarise relevant characteristics of studies.	Yes
**Synthesis of results**	8	Present results for main outcomes, preferably indicating the number of included studies and participants for each. If meta-analysis was done, report the summary estimate and confidence/credible interval. If comparing groups, indicate the direction of the effect (i.e. which group is favoured).	Yes
**DISCUSSION**			
**Limitations of evidence**	9	Provide a brief summary of the limitations of the evidence included in the review (e.g. study risk of bias, inconsistency and imprecision).	Yes
**Interpretation**	10	Provide a general interpretation of the results and important implications.	Yes
**OTHER**			
**Funding**	11	Specify the primary source of funding for the review.	Yes
**Registration**	12	Provide the register name and registration number.	Yes

From: Page MJ, McKenzie JE, Bossuyt PM, Boutron I, Hoffmann TC, Mulrow CD, et al. The PRISMA 2020 statement: an updated guideline for reporting systematic reviews. MetaArXiv. 2020, September 14. DOI: 10.31222/osf.io/v7gm2. For more information, visit: www.prisma-statement.org
